# Kinetic Modeling of the Arabidopsis Cryptochrome Photocycle: FADH^o^ Accumulation Correlates with Biological Activity

**DOI:** 10.3389/fpls.2016.00888

**Published:** 2016-06-28

**Authors:** Maria Procopio, Justin Link, Dorothy Engle, Jacques Witczak, Thorsten Ritz, Margaret Ahmad

**Affiliations:** ^1^Department of Physics and Astronomy, University of California, IrvineIrvine, CA, USA; ^2^UMR 8256 (B2A), IBPS, University of Paris VIParis, France; ^3^Department of Physics, Xavier UniversityCincinnati, OH, USA; ^4^Department of Biology, Xavier UniversityCincinnati, OH, USA

**Keywords:** cryptochrome, flavoprotein, kinetic modeling, signaling, photoreduction

## Abstract

Cryptochromes are flavoprotein photoreceptors with multiple signaling roles during plant de-etiolation and development. *Arabidopsis* cryptochromes (cry1 and cry2) absorb light through an oxidized flavin (FAD_ox_) cofactor which undergoes reduction to both FADH° and FADH^−^ redox states. Since the FADH° redox state has been linked to biological activity, it is important to estimate its concentration formed upon illumination *in vivo*. Here we model the photocycle of isolated cry1 and cry2 proteins with a three-state kinetic model. Our model fits the experimental data for flavin photoconversion *in vitro* for both cry1 and cry2, providing calculated quantum yields which are significantly lower in cry1 than for cry2. The model was applied to the cryptochrome photocycle *in vivo* using biological activity in plants as a readout for FADH° concentration. The fit to the *in vivo* data provided quantum yields for cry1 and cry2 flavin reduction similar to those obtained *in vitro*, with decreased cry1 quantum yield as compared to cry2. These results validate our assumption that FADH° concentration correlates with biological activity. This is the first reported attempt at kinetic modeling of the cryptochrome photocycle in relation to macroscopic signaling events *in vivo*, and thereby provides a theoretical framework to the components of the photocycle that are necessary for cryptochrome response to environmental signals.

## Introduction

Plants adapt to their light environment by means of multiple photoreceptors which optimally absorb at different wavelengths of light throughout the visible spectrum. These include specific photoreceptors absorbing in the blue—UV/A such as cryptochromes (Chaves et al., 2011; Wang et al., 2014) and phototropins (Christie et al., 2015), red/far red light absorbing phytochromes (Burgie and Vierstra, 2014; Xu et al., 2015) and UV-B specific receptors (Jenkins, 2014). Light sensitivity is achieved through pigment molecules (chromophores) bound to a protein backbone (apoprotein). The pigments absorb photons at specific wavelengths of light to initiate a primary photochemical reaction. These reactions, in turn, trigger changes within the photoreceptor apoprotein leading to the initiation of biological signaling. Generally, such changes involve conformational change in the protein which allows access to signaling partners and/or modifications such as phosphorylation or ubiquitination (Galvão and Fankhauser, 2015).

In *Arabidopsis*, two cryptochromes have been shown to mediate significant signaling functions (cry1 and cry2) (Chaves et al., 2011). These proteins are highly conserved within their first 500 amino acid residues, which comprise the N-terminal flavin binding domains. This domain absorbs light and undergoes the primary photochemical reactions involving intra-protein electron and proton transfer to the flavin. By contrast, cry1 and cry2 diverge greatly at their C-terminal domains, which undergo conformational change involved in signaling. Cry1 plays a key role in de-etiolation responses and photomorphogenesis as well as in many aspects of vegetative growth. Cry2 also plays a role in seedling photomorphogenesis, including hypocotyl growth inhibition and cotyledon expansion. However, Cry2 function during de-etiolation is apparent primarily at low blue light intensity and not at high light. This specificity of cry2 for conditions of dim blue light is thought to follow from the fact that cry2, once activated by blue light, undergoes rapid ubiquitination resulting in targeting to the proteosome and degradation (Yu et al., 2007, 2009). In addition to its role in de-etiolation responses, cry2 has been implicated in the *Arabidopsis* photoperiodic initiation of flowering response wherein long days induce earlier flowering than short days (Valverde et al., 2004). Mechanistically, both cry1 and cry2 have been shown to interact with signaling partners (CIB1, SPA1) (Liu et al., 2008, 2011) in response to illumination, indicative of a light induced conformational change leading to substrate binding. In this respect, cryptochrome functions similarly to other classes of known plant photoreceptors (phytochrome, phototropin, and UVR8 type receptors) which also undergo conformational changes in response to illumination.

**Figure 1:**
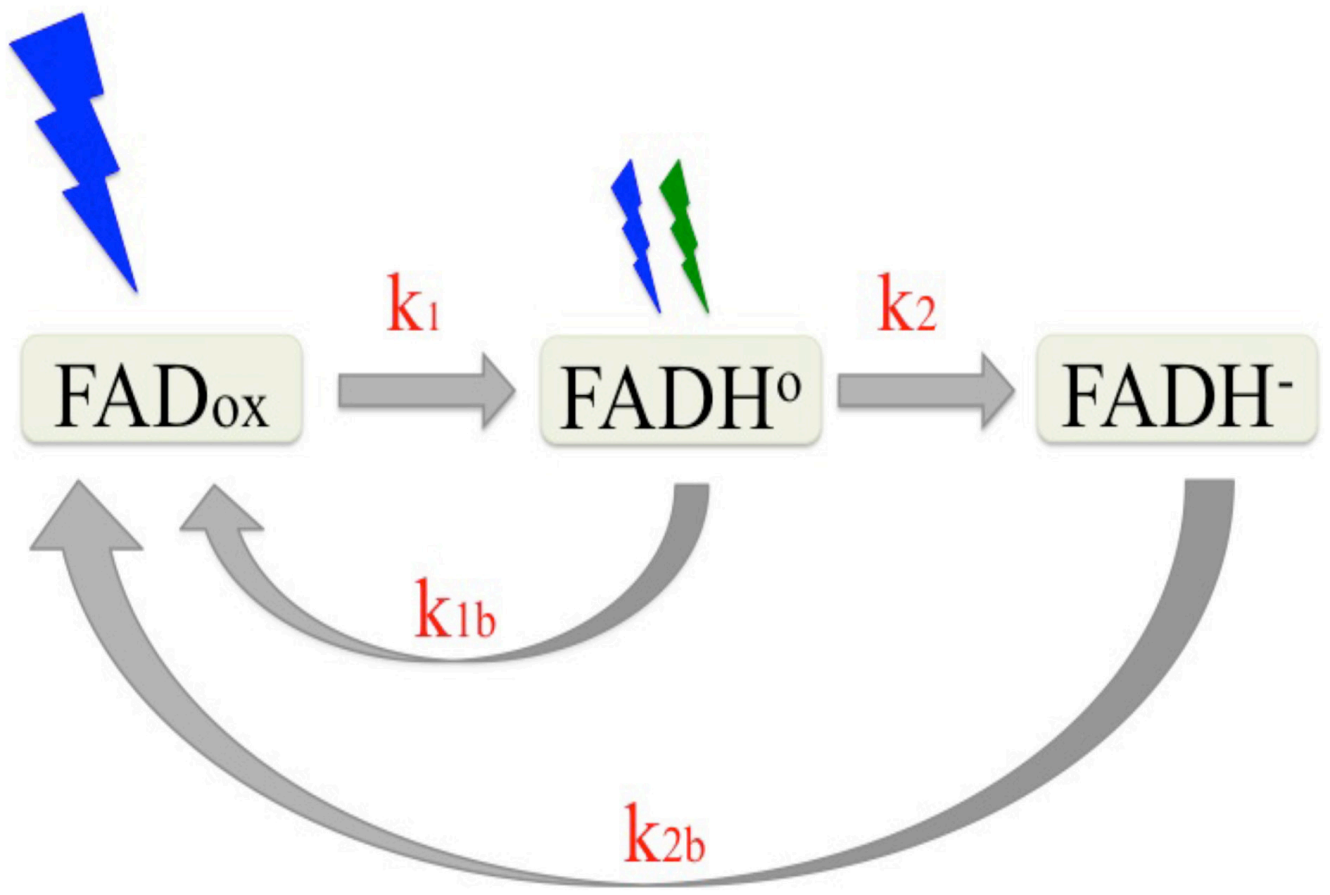
**Reaction scheme of the plant cryptochrome photocycle**. In the dark, flavin occurs in the oxidized (blue light absorbing) redox state (FAD_ox_). Subsequent to illumination, the flavin is photoreduced to the neutral radical (FADH°) redox form by blue light at a defined rate (*k*_1_) determined by the quantum yield. The FADH° can be further reduced to the fully reduced (FADH^−^) form by blue or green light with at a rate *k*_2_. The reduced flavins (FADH° and FADH^−^) reoxidize to the resting state (FAD_ox_) (*k*_1__*b*_, *k*_2__*b*_) at rates that are independent of light. Equilibrium concentrations of the different redox forms of cryptochrome result from the sum of the four reaction rates (*k*_1_, *k*_2_, *k*_1__*b*_, *k*_2__*b*_).

Photochemical reactions of cryptochromes are induced by light absorption through the flavin (FAD) chromophore, and have been well characterized (reviewed in Chaves et al., 2011). Briefly, cryptochrome—bound FAD occurs in the oxidized (FAD_ox_) state in the dark. Upon illumination, the excited state flavin is reduced via multiple electron and proton transfer events to a mixture of neutral radical (FADH°) and fully reduced (FADH^−^) flavin redox states. Once formed, the reduced redox state intermediates are relatively stable (on the order of minutes) and undergo reoxidation to the dark (FAD_ox_) resting state at rates that are determined by the concentration of molecular oxygen (Müller and Ahmad, 2011). Therefore, the proportion of cryptochrome in any given redox state under constant illumination is determined by the steady state equilibrium reached between the forward (light driven photoreduction to FADH° and FADH^−^) and reverse (reoxidation to FAD_ox_) reactions. A description of this redox cycle is shown in Figure 1.

Many lines of evidence have identified the radical (FADH°) redox intermediate is the signaling state (biologically activate) form of cryptochrome. Briefly, the action spectrum for cryptochrome (Ahmad et al., 2002) and *in vivo* measurements of cryptochrome-bound flavin redox state in living cells indicate that the inactive (light-absorbing) state of the photoreceptor is the oxidized (FAD_ox_) redox state (Banerjee et al., 2007; Bouly et al., 2007; Engelhard et al., 2014). Upon illumination, a large conformational change has been shown to occur *in vitro* and linked to formation of the FADH° redox state (Kondoh et al., 2011). The lifetime of this FADH° redox state correlates with that of the biologically active “lit” state for both cry1 and cry2 *in vivo* (Herbel et al., 2013). Significantly, both cry1 and cry2 biological activity is diminished under illumination treatments (green light) that deplete the concentration of the FADH° redox state (see Figure 1; Banerjee et al., 2007; Bouly et al., 2007; Herbel et al., 2013). Finally, in each instance where *in vivo* biological activity has been reported to occur in mutants of cry2 or cry1 in the literature (Li et al., 2011; Gao et al., 2015) the light dependent reduction of the flavin cofactor has also been shown to occur under *in vivo* conditions (Engelhard et al., 2014; El-Esawi et al., 2015). From these and many other studies (see Chaves et al., 2011), the available evidence is consistent with *Arabidopsis* cryptochromes being activated by flavin photoreduction and that the neutral radical (FADH°) redox state represents the “lit” state *in vivo*.

Assigning a given redox form of cryptochrome as the “lit” state raises the challenge of directly linking biological activation to the concentration of this redox form induced upon illumination. This task is further complicated by the fact that, unlike most plant photoreceptors which are converted by light into well-defined “on” and “off” chemical states, cryptochromes in fact exist in three redox states. That is, illumination induces formation of not one but two reduced states (FADH° and FADH^−^), each with a characteristic (and different) reoxidation rate that occurs continuously throughout the illumination period. Therefore, the concentration of the “activated” redox state results from the equilibrium defined by the sum rate constants (*k*_1_, *k*_2_, *k*_1__*b*_, *k*_2__*b*_) that must be considered as a function of the quality (wavelength) and intensity of the light signal.

In this study, we fit a three-state kinetic model of cryptochrome photocycle to absorption spectra of isolated proteins illuminated *in vitro*. We use the kinetic model to obtain the rate constants (Figure 1) and calculate the quantum yield of the light-driven reactions (FAD_ox_ to FADH° and FADH° to FADH^−^). To apply the kinetic model to cryptochrome responses *in vivo*, it is not possible in living plants to directly measure the concentration of the different redox state intermediates. We therefore consider biological activity as a measure for FADH° concentration formed *in vivo*. Indeed, the experimental data for cryptochrome responses *in vivo* at different intensities and wavelengths of light provided an excellent fit for the kinetic model applied to spectra *in vitro*. This model thereby validates the assumption that FADH° is the signaling state, and provides further insight into many of the known characteristics of cryptochrome responses *in vivo*.

## Materials and methods

### Cryptochrome protein samples

Arabidopsis cryptochrome-1 (cry1) and cryptochrome-2 (cry2) proteins were expressed and purified using baculovirus expression constructs in insect cell cultures as previously described (Banerjee et al., 2007; Bouly et al., 2007). Photoreduction experiments were conducted by illuminating protein samples in PBS (Phosphate Buffered Saline: 5 mM NaPO_4_ pH 7.5 or 8.2, 150 mM NaCl directly in quartz cuvettes, 1 cm path length) at 21°C. Spectra were taken using a Cary 300 UV/Vis spectrophotometer at 21°C. Concentration of cry1 and cry2 protein samples was between 50 and 150 μM for *in vitro* experiments.

### Light sources

LEDS were from Quadica Developments, Brantford, Ontario. Blue light: Royal-Blue (447.5 nm). Green Light: Lime-Green (567 nm). Spectra are as indicated by the manufacturer.

### Western blotting

Seeds from *phyAphyB Arabidopsis* phytochrome-deficient mutants from ecotype Landsberg erecta (Ahmad and Cashmore, 1997) were sown and germinated as described previously (Herbel et al., 2013). Germinating seedlings were maintained for four days at 22°C in darkness. Etiolated seedlings were then illuminated, harvested into liquid nitrogen, the proteins extracted and then applied to nitrocellulose membranes for Western blotting as described previously (Herbel et al., 2013). Quantitation of the cry2 signal from the Western blots was by ImageJ image analysis software from photographic images of the blots.

### Kinetic model

The reaction scheme depicted in Figure 1 reports the cryptochrome photocycle studied here. The time evolution of the intermediate states concentrations is described by a set of coupled first-order kinetic equations (Espensen, 1981):

(1) $$\begin{array}{l} \left\{ \begin{array}{l} \frac{d\left[FAD_{ox}\right]}{dt}\;=-k_{1}\left[FAD_{ox}\right]+\;k_{1b}\left[FADH^{o}\right]+\;k_{2b}\left[FADH^{-}\right]\\ \frac{d\left[FADH^{o}\right]}{dt}=k_{1}\left[FAD_{ox}\right]-\left(k_{2}\;+\;k_{1b}\right)\left[FADH^{o}\right]\\ \frac{d[FADH^{-}]}{dt}\;=k_{2}\left[FADH^{o}\right]-k_{2b}\left[FADH^{-}\right] \end{array}\right. \end{array}$$

where *k*_1_ and *k*_2_ are the two forward rate constants, and *k*_1__*b*_ and *k*_2__*b*_ are the dark reoxidation rate constants. In Equation (1) square brackets denote the concentrations of the transient states FAD_ox_, FADH^o^, and FADH^−^. The fit of the kinetic model to absorption spectra of the isolated proteins allows to find the rate constants. To this end we apply the Beer-Lambert law, which relates the concentration of the transient states to the absorbance *A* (Schmidt, 2005). According to the Beer-Lambert law the absorbance *A* at a given wavelength λ and at time *t* is linearly dependent on the concentration of the absorbing species:

(2) $$\begin{array}{l} A\left(\lambda ,t\right)=d\sum_{i=1}^{N}\varepsilon_{i}\left(\lambda \right)c_{i\;}\left(t\right) \end{array}$$

where N is the number of different light-absorbing species in the system with concentration *c*_*i*_ (M). ε_*i*_ (M^−1^ cm^−1^) is the molar extinction coefficient and *d* (cm) is the thickness of the absorbing medium. In all our experiments we use the same quartz cuvette of path length *d* = 1 cm.

We record absorption spectra from 400 to 570 nm, by illuminating cry samples with blue (450 ± 10 nm) and green light (560 ± 10 nm). The neutral radical (FADH°) flavin redox state can absorb green as well as blue light, while the FAD_ox_ absorbs blue (Banerjee et al., 2007; Bouly et al., 2007). The FADH^−^ radical absorbs at wavelengths out of the range considered here (Müller and Ahmad, 2011). Thus, from the Beer-Lambert law we have:

(3) $$\begin{array}{lll} \text{A}\left(450,t\right) & =\varepsilon_{ox}\left(450\right)\left[{\text{FAD}}_{\text{ox}}\right]\left(t\right)+\;\varepsilon_{H}\left(450\right)\left[{\text{FADH}}^{\text{o}}\right]\left(t\right)\; & \;\\ \text{A}\left(560,t\right) & =\varepsilon_{H}\;\left(560\right)\left[{\text{FADH}}^{\text{o}}\right]\left(t\right)\; & \; \end{array}$$

where ε_*ox*_(450) and ε_*H*_(560) are the molar extinction coefficients, respectively, of FAD_ox_ and FADH^o^.

Since cry–bound FAD only occurs in the oxidized (FAD_ox_) state in the dark, the initial concentration of cry in the sample before illumination, i.e., at time *t* = 0, can be found according to *A*(450, 0) = ε_*ox*_(450)[FADox](0), while the other intermediate states are unpopulated.

Based on the absorption spectra of FAD_ox_ and FADH^o^ (Liu et al., 2010; Björn, 2015) we estimate that the extinction coefficient of FADH^o^ at 450 nm and 560 nm is the same and equals ε_*H*_ (450) = ε_*H*_ (560) = ε_*ox*_ (450)/2.

By normalizing absorbance and concentration to the dark, and considering that ε_*H*_ (450) = ε_*ox*_ (450)/2, and ε_*H*_ (560) = ε_*ox*_ (450)/2, we obtain from Equation (3) a simplified expression between concentrations and absorbance, which we use in our calculations:

(4) $$\begin{array}{lll} \left[{FAD}_{ox}\right]\left(\text{t}\right) & =A\left(450,t\right)-A\left(560,t\right)\; & \;\\ \left[{FADH}^{o}\right]\left(\text{t}\right) & =2A\left(560,t\right)\; & \; \end{array}$$

In Equation (4) square brackets and *A* label, respectively, normalized concentration and normalized absorbance. We numerically solve Equation (1) with a Runge-Kutta method, and obtain the time evolution of the concentration of the transient states corresponding to data acquisition times, with the assumption that at a given time *t*, the sum of the concentrations of the three states is constant, i.e., $\left[{FAD}_{ox}\right]\left(t\right)\;+\;\left[{FADH}^{o}\right]\left(t\right)\;+\;\left[{FADH}^{-}\right]\left(t\right)=1.$

#### Forward rates

In Equation (1), while reoxidation rates *k*_1__*b*_ and *k*_2__*b*_ are independent of light, the forward rates *k*_1_ and *k*_2_ are light dependent. At a given wavelength λ the rate constant is given by *k*_λ_ = σ_λ_ *I*_λ_ where *I*_λ_ is the photon fluence rate (mol m^−2^ s^−1^) at wavelength λ and σ_λ_ (mol^−1^ m^2^) denotes the photoconversion cross-section. σ_λ_ is related to the quantum yield ϕ_λ_ according to σ_λ_ = 2.3 ε(λ) ϕ_λ_ (Kendrick and Kronenberg, 1994).

In our model we consider that the efficiency of photoconversion of FADH^o^ upon blue light illumination is negligible (see Section Results). Thus the forward rates *k*_1_ and *k*_2_ are related to the photon fluence rate of blue light *I*_1_ and of green light *I*_2_ by, respectively, *k*_1_ = σ_1_ *I*_1_ and *k*_2_, *k*_2_ = σ_2_*I*_2_. *I*_2_, where ε_1_ = ε_*ox*_(450) and ε_2_ = ε_*H*_(560).

#### Two-state model: dark reoxidation *k*_1*b*_, and quantum yield ϕ_1_ under blue light

In order to determine the rate constants *k*_1_ and *k*_1__*b*_, we first consider an abbreviated photocycle consisting of two states FAD_ox_ and FADH^o^. This is possible because in atmospheric oxygen and in the presence of mild reductants, isolated *Arabidopsis* cry1 and cry2 proteins, under blue light illumination, accumulate in primarily two redox forms: FAD_ox_ and FADH° (Banerjee et al., 2007; Bouly et al., 2007). The two-state kinetic model is described by the following system of differential equations:

(5) $$\begin{array}{l} \{\begin{array}{c} \frac{d[FAD_{ox}]}{dt}\;=-k_{1}\left[FAD_{ox}\right]+\;k_{1b}[FADH^{o}]\\ \frac{d[FADH^{o}]}{dt}=k_{1}\left[FAD_{ox}\right]-k_{1b}[FADH^{o}] \end{array} \end{array}$$

We set the initial concentration of cry at time *t* = 0, i.e., before illumination to [FADox](0) = 1. Furthermore, at a given later time *t* $\left[{FAD}_{\text{ox}}\right]\left(t\right)\;+\;\left[{FADH}^{o}\right]\left(t\right)=1$. Analytical solutions of Equation (5) are straightforward (see Equations S1, S2 in Supplementary Material).

##### Dark reoxidation rate (k_1*b*_)

Since flavin reoxidation from FADH° to FAD_ox_ can be readily monitored spectroscopically, we first obtain experimental values for the dark reoxidation kinetics from reduced (FADH°) to oxidized (FAD_ox_) flavin states for the cry.

We define the reoxidation time as *t*_*d*_, and *t*_*d*_ = 0 is the time in which cry is placed in darkness after being illuminated for a certain time t at fluence rate *I*_1_. We record the absorption spectrum after increasing times in darkness *t*_*d*_, until complete reoxidation to FAD_ox_. When only the dark reoxidation occurs (*k*_1_ = 0) the analytical solutions of Equation (2) are:

(6) $$\begin{array}{lll} \;\left[\;{FAD}_{ox}\right]\left(t_{d}\right) & =c_{ox}\;+\;c_{o}\left(1-e^{-k_{1b}t_{d}}\right)\; & \; \end{array}$$

(7) $$\begin{array}{lll} \left[{FADH}^{o}\right]\left(t_{d}\right) & =c_{o}e^{-\left(k_{1b}t_{d}\right)}\; & \; \end{array}$$

where *c*_*ox*_ and *c*_*o*_ are the initial concentrations of, respectively, FAD_ox_ and FADH^o^ at the dark time *t*_*d*_ = 0. We plot the concentrations of FAD_ox_ and FADH^o^, obtained from the spectra by applying Equation (4), as a function of the dark recovery time t_d_, and fit the data with, respectively, Equations (6) and (7) to find *k*_1__*b*_. For the fitting model we use the Least-Squares algorithm Levenberg-Marquardt provided by Matlab. We calculate the half-life τ_1__∕2_ that, for a first-order reaction, is giving by τ_1∕2_ = ln(2) $k_{1b}^{-1}$.

##### Forward photoreduction rate (k_1_). two-state-based algorithm

For an illumination time much smaller than the reoxidation time *k*_1*b*_ = 0 in Equation (2). The analytical solutions for this case can be used to calculate the rate constant *k*_1_ from the absorption spectra (see Equations S3, S4 in Supplementary Material). However, for longer illumination times, the reoxidation rate has to be taken into account. For this we implement a simple algorithm based on Equation (5). Such algorithm takes as input either the [FAD_ox_] or [FADH^o^] concentration, the *k*_1*b*_ previously found, and outputs the corresponding rate constant *k*_1_ according to the two-state kinetic model. The concentration of FAD_ox_ and FADH^o^ are obtained from the spectra by using Equation (4). In our calculations we use as input both [FAD_ox_] and [FADH^o^] concentration and compare the results to confirm that the two-state model approximates well the blue-light experiments.

##### Quantum yield of FAD_*ox*_—FADH° conversion

We calculate the quantum yield ϕ_1_ from the photoconversion cross section σ_1_ according to σ_1_ = 2.3 ε_*ox*_(450) ϕ_1_ (Kendrick and Kronenberg, 1994). To obtain the photoconversion cross section we illuminate cry sample with increasing blue light fluence rates *I*_1_ and record absorption spectra. For each photon fluence rate *I*_1_ we calculate *k*_1_ with the two-state algorithm described above. Plotting *I*_1_ vs. *k*_1_, and fitting the data with a linear function, *k*_1_ = σ_1_ *I*_1_, allows estimation of σ_1_. From σ_1_ we calculate ϕ_1_ by using the experimentally determined extinction coefficient of cry1 and cry2 (see Section Extinction Coefficient). We point out that only one single photon fluence rate value is enough to calculate the quantum yield. However, we prefer to use a dose-response profile to find a linear range of blue light fluence rates that both allows prediction, and confirms the linear correlation between *k*_1_ and *I*_1_.

#### Three-state model: forward rate *k_2_* and quantum yield ϕ_2_ under green light

##### Forward photoreaction rate k_2_. three-state-based algorithm

To find the rate constant *k*_2_ we co-illuminate samples with blue and green light at fluence rates, respectively, *I*_1_ and *I*_2_, and record the absorption spectrum. From the spectra we obtain the normalized concentration of [FAD_ox_] and [FADH^o^] by using Equation (4). To calculate *k*_2_ we implement an algorithm by numerically solving Equation (1). This algorithm takes as input the concentration of either [FAD_ox_] or [FADH^o^], *k*_1_, *k*_1*b*_ and *k*_2*b*_, and outputs *k*_2_, according to the three-state model. We use the *k*_1_ and *k*_1*b*_ values obtained from the blue light experiments in the present study, and *k*_2*b*_ = 0.011 s^−1^ is provided from previous studies (Müller and Ahmad, 2011). For short illumination times, one can neglect reoxidation rates, and the algorithm in this case resolves Equation (1) with *k*_1*b*_ = *k*_2*b*_ = 0.

##### Quantum yield of FADH^*o*^—FADH^−^ conversion

We calculate the quantum yield ϕ_2_ from the photo-conversion cross section σ_2_ according to σ_2_ = 2.3 ε_2_ ϕ_2_. To this end we perform a series of experiments to obtain a dose-response profile. We illuminate cry samples with same blue light fluence rate *I*_1_ and increasing green light fluence rates *I*_2_. For each photon fluence rate *I*_2_ we calculate the corresponding *k*_2_ by using the three-state algorithm. We plot *I*_2_ vs. *k*_2_ and fit the data with a linear function, *k*_2_ = σ_2_ *I*_2_, to estimate the photo-conversion cross section σ_2_. We then calculate ϕ_2_ by considering that ε_2_ = ε_*ox*_(450)/2.

#### Extinction coefficient

We calculated the extinction coefficient at 450 nm of cry1 and cry2 using absorption of the purified cryptochrome protein at 450 nm together with protein concentration determined by Bradford assay. The extinction coefficient of cry1 resulted ε_*ox*_ (450) = 6415.5 M^−1^ cm^−1^, and that of cry2 ε_ox_ (450) = 5094 M^−1^ cm^−1^. see Supplementary Figure A. In our calculations we use as units for the extinction coefficient mol^−1^ m^2^, thus for cry1 ε_ox_(450) = 641.55 mol^−1^ m^2^, and for cry2 ε_*ox*_(450) = 509.4 mol^−1^ m^2^.

### Kinetic model applied to *in vivo* responses

To apply the kinetic model to *in vivo* responses we make the assumption that biological activity is directly proportional to the concentration of the FADH° flavin state (Banerjee et al., 2007; Bouly et al., 2007). In the case of cry1, we choose blue-light dependent inhibition of hypocotyl elongation as a “readout” for biological activity, and consider the concentration of FADH° as inversely proportional to the blue-light dependent inhibition of hypocotyl length (L). In the case of cry2 we use light-dependent degradation of cry2 as a readout for biological function, and we consider the concentration of FADH° as inversely proportional to the cry2 protein concentration (C). In both cases, phytochrome-deficient *phyAphyB* mutants were used for assay of cry-dependent function, in order to avoid the considerable effect of phytochrome, which also absorbs in the blue and green spectral regions and significantly enhances the sensitivity of cryptochrome-dependent signaling pathways (Ahmad and Cashmore, 1997). Therefore, in our studies, only the effects of light on the cry (blue light receptor) are detected as biological activity.

The length L or concentration C are measured as a function of the photon fluence rate, thereby obtaining a dose-dependent biological response profile. We convert this light dose-biological response curve into a light dose-FADH^o^ concentration curve by using the Equation (S5) given in Supplementary Material. In this way we can calculate quantum yields by applying the same method as for *in vitro* data.

#### Two-state model. quantum yield of FAD_ox_—FADH° conversion

For each blue light fluence rate *I*_1_, we calculate the rate constant *k*_1_ by the two-state algorithm, which takes as input the FADH^o^ values and the dark reoxidation *k*_1*b*_ provided from previous studies (Herbel et al., 2013). By plotting *I*_1_ vs. *k*_1_, and fitting the data with expression *k*_1_ = σ_1_ *I*_1_, we estimate the photoconversion cross section σ_1_. We calculate the quantum yield ϕ_1_ according to σ_1_ = 2.3 ε_1_ ϕ_1_ and by using the experimentally determined extinction coefficient ε_1_ = ε_ox_(450) found for cry *in vitro*.

#### Three-state model

Seedlings were co-illuminated with a blue light fluence rate *I*_1_, and increasing fluence rates of green light *I*_2_. A dose-biological response profile was then converted in dose-FADH^o^ profile. For each photon fluence rate *I*_2_ we calculate the rate constant *k*_2_ by the three-state-based algorithm, which inputs FADH^o^, *k*_1_ previously obtained with the blue light experiments, and *k*_1*b*_ and *k*_2*b*_ provided from the literature (Müller and Ahmad, 2011; Herbel et al., 2013). We fit the data with a linear function, *k*_2_ = σ_2_ *I*_2_, to estimate σ_2_ and calculate the quantum yield ϕ_2_ using the *in vitro* extinction coefficient ε_2_.

## Results

The goal of this study is to apply a simple kinetic model to the cryptochrome photocycle (Figure 1) that can accurately predict the effects of illumination on redox state interconversion *in vitro* and relate this model to observations on biological activation *in vivo*. We first apply the model *in vitro*, to samples of purified isolated cryptochrome (cry1 and cry2) which were photoreduced under defined illumination conditions. In this way, concentrations of redox state intermediates could be accurately determined and the reaction rates and quantum yields calculated by the model. We next apply the kinetic model to plant cryptochrome responses *in vivo* to correlate flavin redox state interconversion that could account for biological activity.

### Two-state model for cryptochrome photocycle *in vitro*

For analysis of the cryptochrome photocycle *in vitro*, samples of purified cry1 and cry2 proteins were photoreduced *in vitro* and allowed to reoxidize in monochromatic blue light (450 nm). The simpler two-state model (an abbreviated photocycle from FADox to FADH° and back) is valid under these conditions as there is almost no FADH^−^ accumulation (Banerjee et al., 2007; Bouly et al., 2007). We therefore first modeled only the rates *k*_1_ and *k*_1*b*_, and used them to obtain quantum yield and half-life of cry under conditions of monochromatic blue light.

#### Dark reoxidation rate (k_1*b*_)

**Figure 2:**
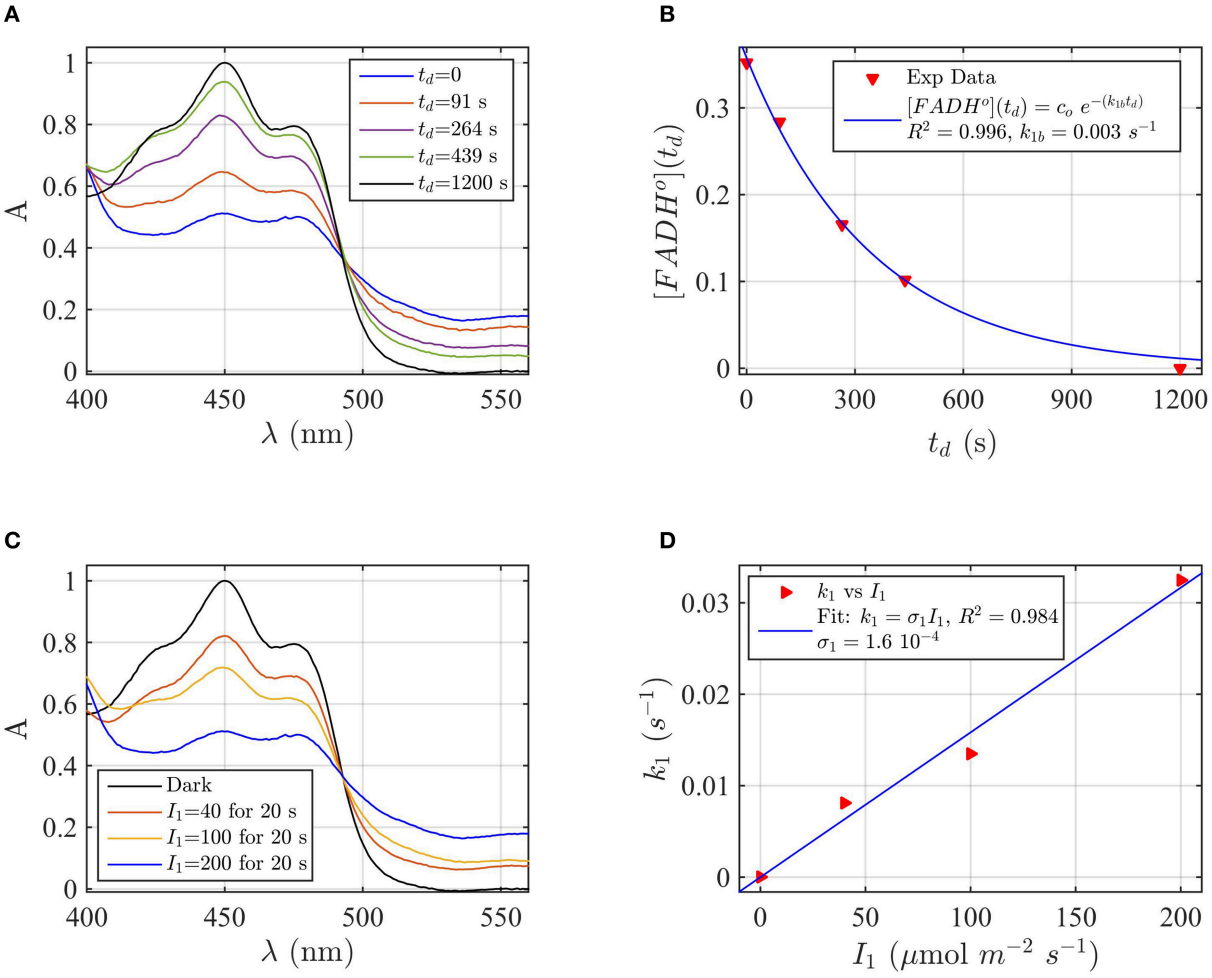
**Rate constants and quantum yield for two-state reduction and reoxidation of AtCry2 in PBS pH7.5, 10 mM βME. (A)** Isolated purified cry2 protein was illuminated for 20 seconds (s) at 200 μmol m^−2^ s^−1^ blue light and placed in darkness (t_d_ = 0 s). Normalized absorption spectra are reported at increasing dark intervals. The FAD_ox_ concentration is obtained from the absorbance at 450 and 560 nm, and FADH^o^ concentration from absorbance at 560 nm according to Equation 4. **(B)** Normalized concentration of FADH^*o*^ as a function of the dark reoxidation time (t_d_). The FADH^o^ concentration is obtained from the spectra of panel **(A)**. The red triangles represent the experimental data, and the blue curve is the fit of the experimental data with the two-state dark reoxidation model for FADH^o^ (Equation 7). The calculated reoxidation rate is *k*_1*b*_ = 0.003 s^−1^ (half-life of τ_1∕2_ = 231 s). The goodness of the fit is large (*R*^2^ = 0.996). **(C)** Isolated purified cry2 protein was illuminated for 20 s at the indicated blue light fluence rates $I_{1},\left(\mu \text{mol}{\text{m}}^{-2}{\text{s}}^{-1}\right)$. Normalized absorption spectra are presented. **(D)** Calculated forward rate constant *k*_1_ vs. photon fluence rate *I*_1_ (red triangles). For each *I*_1_, the rate constant *k*_1_ was calculated by using the two-state algorithm, which inputs the concentration of FADH^o^, obtained from the spectra of panel **(C)**, and *k*_1*b*_ from panel **(B)** and outputs the rate constant *k*_1_. The linear fit *k*_1_ = σ_1_ *I*_1_ of the data are reported in blue. The photo-conversion cross section resulted σ_1_ = 1.6 × 10^−4^ μmol^−1^ m^2^. σ_1_ is related to the quantum yield ϕ_1_ and the extinction coefficient ε_1_ according to σ_1_ = 2.3 ε_1_ ϕ_1_. By using the experimentally calculated ϵ_1_ = 509.4 mol^−1^ m^2^ (5094 M^−1^ cm^−1^) the quantum yield was ϕ_1_ = 0.137. For details of calculations see Method Section: Two-State Model: Dark Reoxidation k_1b_, and Quantum Yield ϕ_1_ under Blue Light.

A sample of Cry2 (pH = 7.5, with 10 mM Betamercaptoethanol as reducing agent) was illuminated with blue light for 20 s at a fluence rate of *I*_1_ = 400 μmol m^−2^s^−1^, and then placed in darkness (*t*_*d*_ = 0). Figure 2A shows the absorption spectrum after increasing times in darkness *t*_*d*_, until complete reoxidation to FAD_ox_. From the spectra of Figure 2A we obtained the concentrations of FAD_ox_ and FADH^o^ as a function of the reoxidation time *t*_*d*_ by using Equation (4). Figure 2B (the red triangles) reports the concentration of FADH^o^ as a function of the dark recovery time *t*_*d*_, and the fit (blue curve) of the data with the two-state dark reoxidation model reported in Equation (7). The fit resulted in a reoxidation rate of *k*_1*b*_ = 0.003 s^−1^, or half-life of τ_1∕2_ = 231 s. We have also fit the increase of FAD_ox_ with Equation (6) and obtained a similar reoxidation rate (*k*_1*b*_ = 0.0036 s^−1^, τ_1∕2_ = 192 s with a goodness of the fit *R*^2^≈1, confirming that the two-state model well approximates our experiments. The average of the two half-lives is reported in Table 1.

**Table 1:** *****In vitro*** and ***in vivo*** parameters of the cry1 and cry2 photocycle depicted in Figure 1**.

	**τ_1∕2_(s)**	**σ_1_(mol^−1^ m^2^)**	**σ2(mol^−1^ m^2^)**	**ϕ_1_**	**ϕ_2_**
***IN VITRO***					
Cry2 pH = 7.5	210	220.0	16.0	0.19	0.027
Cry2 pH = 8.2	187	47.6		0.04	
Cry2 DTT	124	213.0		0.18	
Cry1 DTT	48	56.6		0.038	
***IN VIVO***					
Cry1	300	20.8		0.014	
Cry2	960	480.0	30.0	0.41	0.05

**Figure 3:**
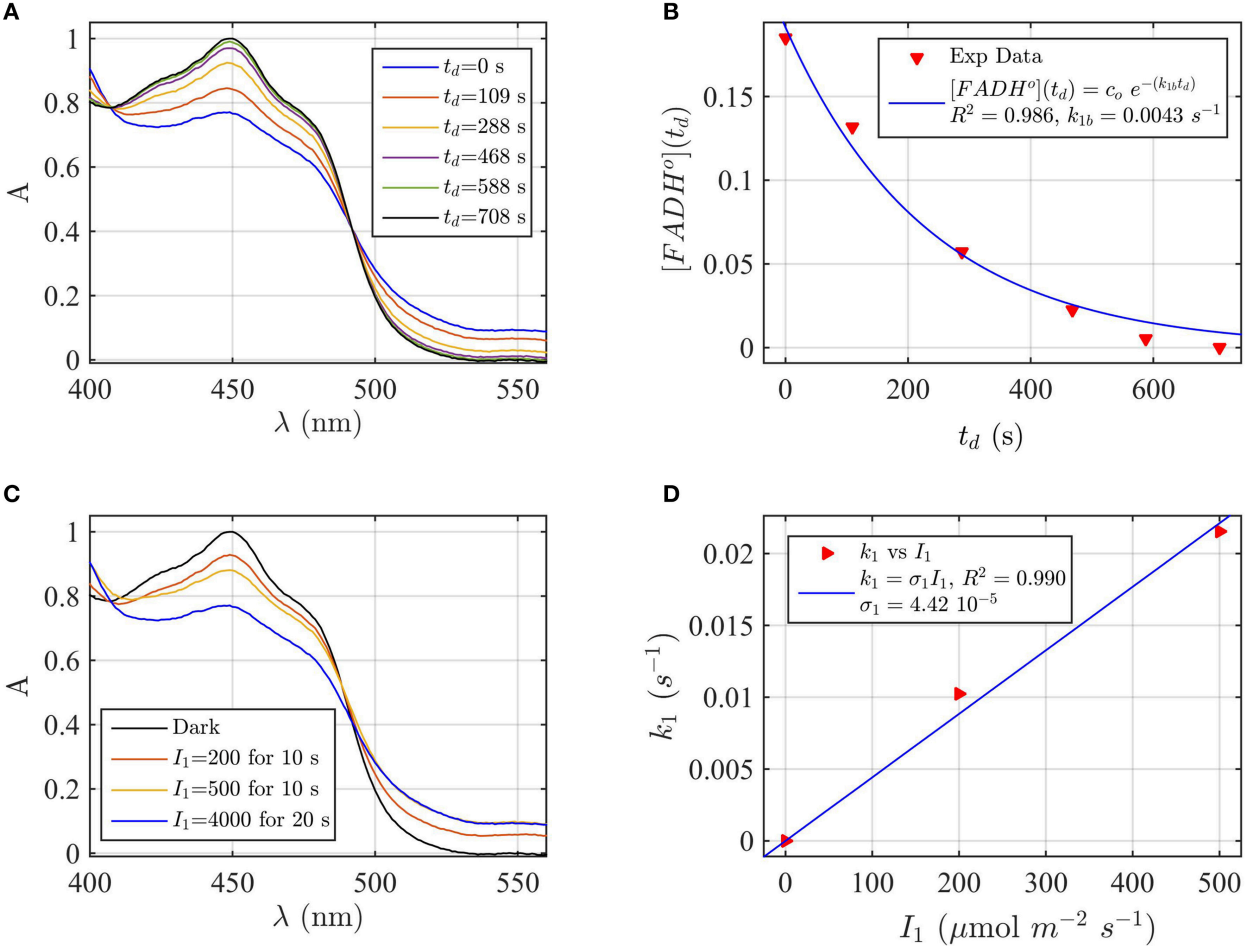
**Rate constants and quantum yield for two-state reduction and reoxidation of AtCry2 in PBS pH 8.2, 10 mM βME. (A)** Isolated purified cry2 protein was illuminated for 20 s at 4000 μmol m^−2^s^−1^ blue light and placed in darkness (t_d_ = 0 s). Normalized absorption spectra are reported at increasing dark intervals. **(B)** Normalized concentration of FADH^o^ as a function of the dark reoxidation time (t_d_). The FADH^o^ concentration is obtained from the spectra of panel **(A)**. The red triangles represent the experimental data, and the blue curve is the fit of the experimental data with the two-state dark reoxidation model for FADH^o^ (Equation 7). The resulting reoxidation rate was *k*_1*b*_ = 0.0043 s^−1^ (half-life of τ_1∕2_ = 2.7 min). **(C)** Isolated purified cry2 protein was illuminated for 10 s at the indicated blue light fluence rates $I_{1},\left(\mu \text{mol}{\text{m}}^{-2}{\text{s}}^{-1}\right)$. Normalized absorption spectra are presented. **(D)** Calculated forward rate constant *k*_1_ vs. photon fluence rate *I*_1_ (red triangles). For each fluence rate *I*_1_ we calculated the rate constant *k*_1_ by using the two-state model algorithm, with input of FADH^o^ (from panel **C**) and *k*_1*b*_ from panel **(B)**. The blue curve reports the linear fit of the data (*k*_1_ = σ_1_*I*_1_), which gives a photoconversion cross section of σ_1_ = 4.42 × 10^−5^ μmol^−1^ m^2^, and a quantum yield of ϕ_1_ = 0.038. For details of calculations see caption of Figure 2.

**Figure 4:**
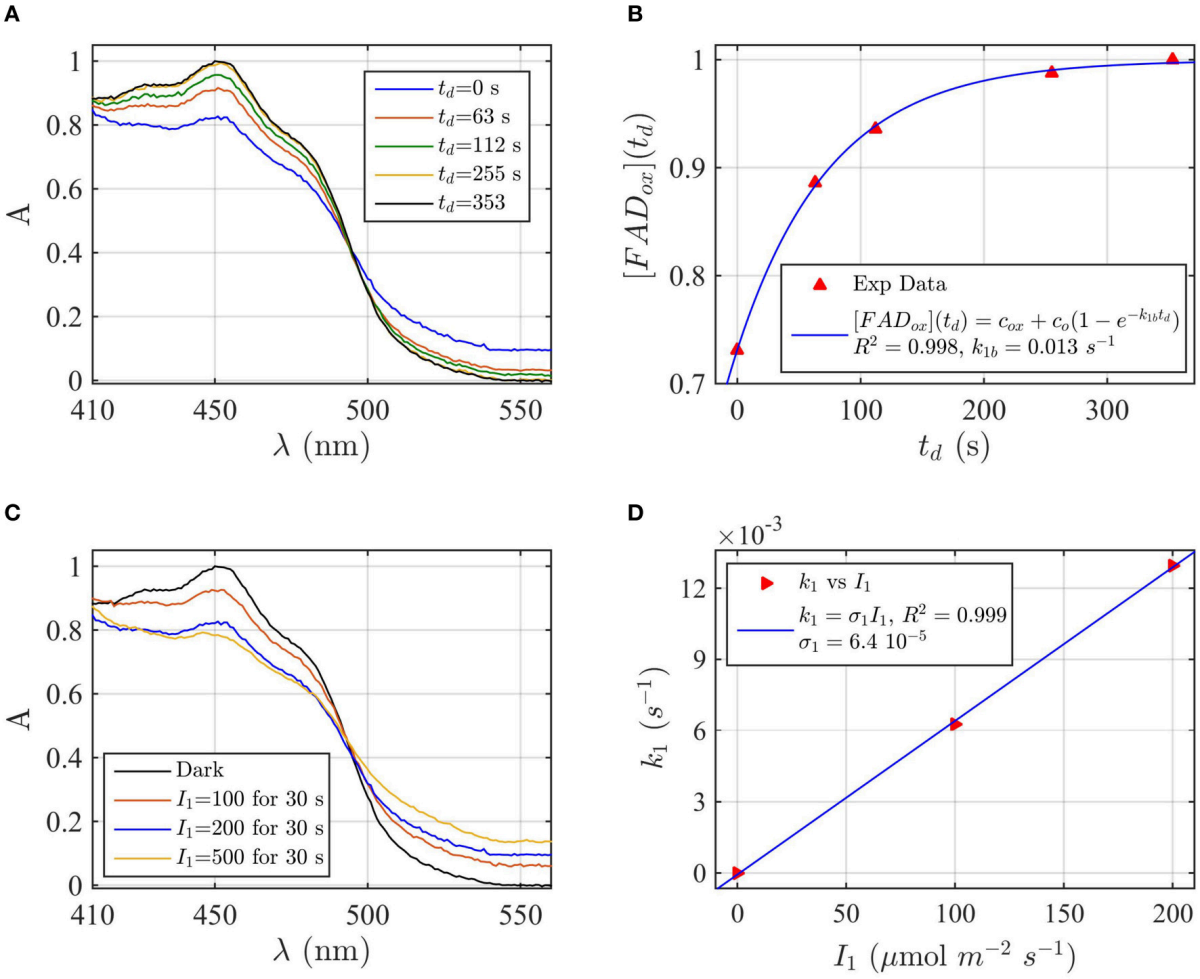
**Rate constants and quantum yield for two-state reduction and reoxidation of AtCry1 in 5 mM DTT. (A)** Isolated purified cry1 protein was illuminated for 30 s at 200 μmol m^−2^s^−1^ blue light and placed in darkness (t_d_ = 0 s). Normalized absorption spectra are shown at increasing dark intervals. **(B)** Normalized concentration of FAD_ox_ as a function of the dark reoxidation time (t_d_). The FAD_ox_ concentration is obtained from the spectra of panel **(A)**. The red triangles represent the experimental data, and the blue curve is the fit of the experimental data with Equation (6). The reoxidation rate resulted *k*_1*b*_ = 0.013 s^−1^ (half-life of τ_1∕2_ = 53 s). **(C)** Isolated purified cry1 protein was illuminated for 30 s at the indicated blue light fluence rates $I_{1},\left(\mu \text{mol}{\text{m}}^{-2}{\text{s}}^{-1}\right)$. Normalized absorption spectra are presented. **(D)** Calculated forward rate constant *k*_1_ vs. photon fluence rate *I*_1_ (red triangles). For each *I*_1_ of panel **(C)**, the rate constant *k*_1_ was calculated by using the two-state model algorithm with concentration of FAD_ox_ and *k*_1*b*_ (from panel **B**) as input. From the linear fit of the data, *k*_1_ = σ_1_ *I*_1_ (shown in blue) the photoconversion cross section resulted in σ_1_ = 6.4 × 10^−5^ μmol^−1^ m^2^, which gives a quantum yield of ϕ_1_ = 0.043. For details of calculations see caption of Figure 2.

**Figure 5:**
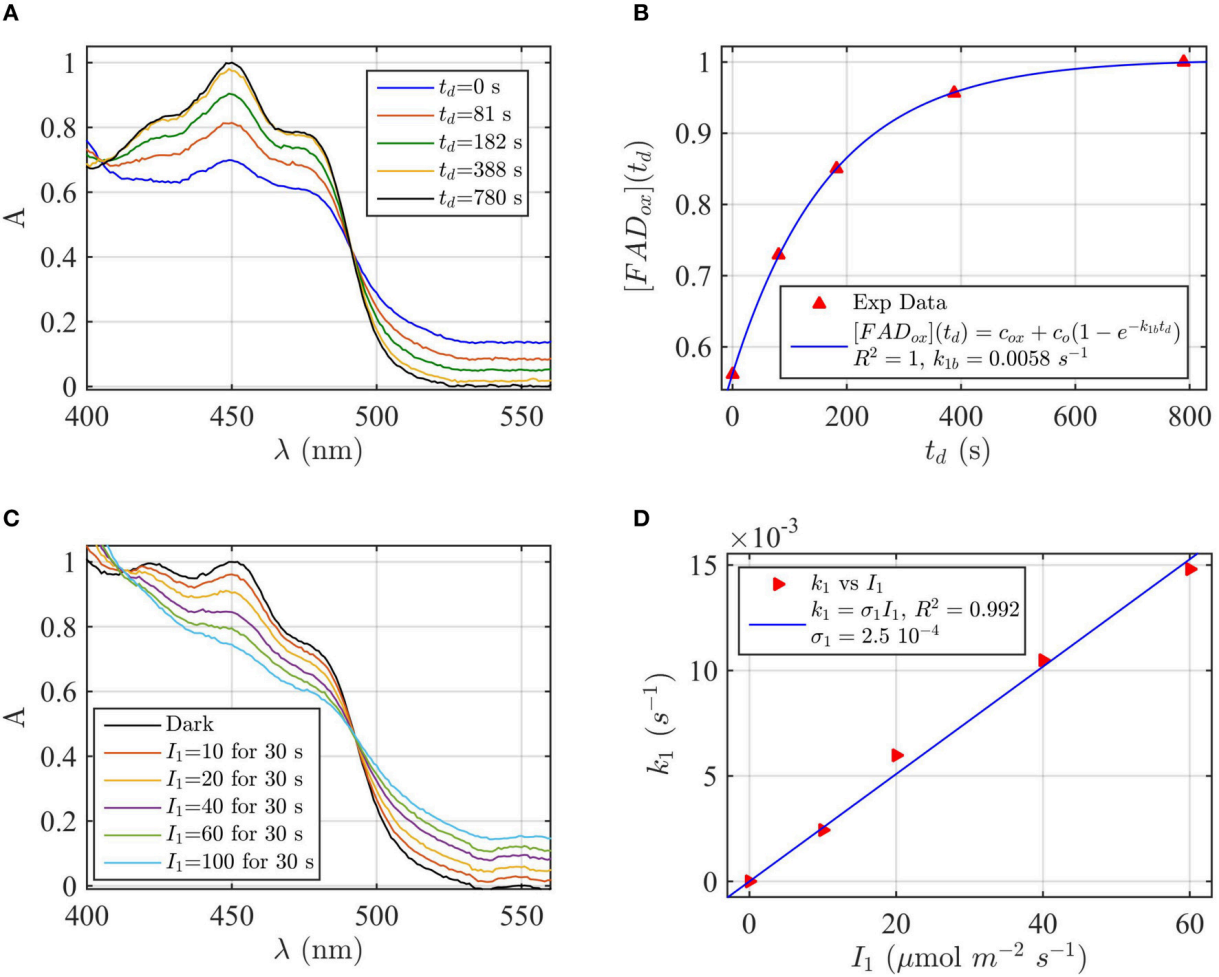
**Rate constants and quantum yield for two-state reduction and reoxidation of AtCry2 in 5 mM DTT. (A)** Isolated purified cry2 protein was illuminated for 30 s at 200 μmol m^−2^s^−1^ blue light and placed in darkness (t_d_ = 0 s). Normalized absorption spectra are reported at increasing dark intervals. **(B)** Normalized concentration of FAD_ox_ as a function of the dark reoxidation time (t_d_). The FAD_ox_ concentration is obtained from the spectra of panel **(A)**. The red triangles represent the experimental data, and the blue curve is the fit of the experimental data with Equation (6). The reoxidation resulted *k*_1*b*_ = 0.0058 s^−1^ (half-life of τ_1∕2_ = 2 min). **(C)** Isolated purified cry2 protein was illuminated for 30 s at the indicated blue light fluence rates $I_{1},\left(\mu \text{mol}{\text{m}}^{-2}{\text{s}}^{-1}\right)$. Normalized absorption spectra are presented. **(D)** Calculated forward rate constant *k*_1_ vs. photon fluence rate *I*_1_ (red triangles). For each fluence rate *I*_1_ of panel **(C)**, the rate constant *k*_1_ was calculated by using the two-state algorithm, with FAD_ox_ concentration and *k*_1*b*_ from panel **(B)** as input. From the linear fit of the data *k*_1_ = σ_1_ *I*_1_ (shown in blue) the photoconversion cross section resulted σ_1_ = 2.5 × 10^−4^ μmol^−1^ m^2^, which gives a quantum yield of ϕ_1_ = 0.213. For details of calculations see caption of Figure 2.

**Figure 6:**
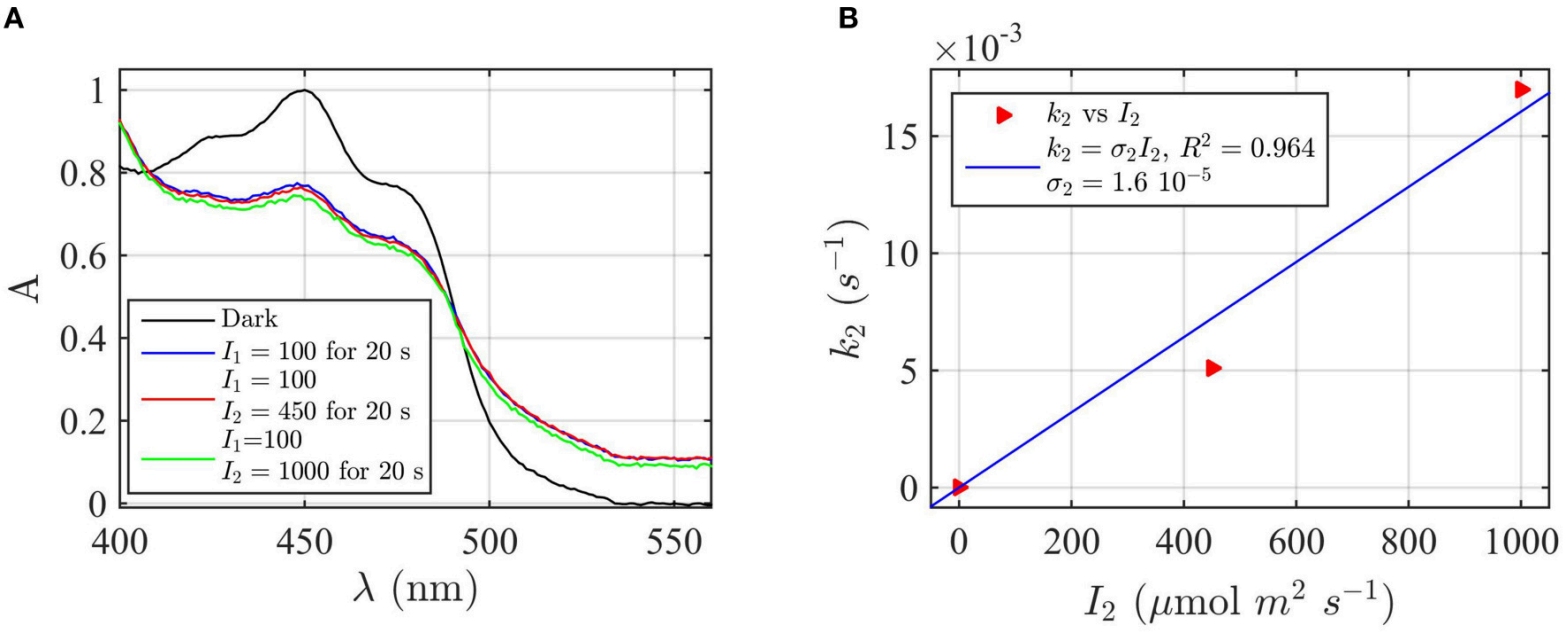
**Forward rate constant ***k***_**2**_ and quantum yield ϕ_**2**_ under green light for AtCry2 in PBS pH7.5, 10 mM βME. (A)** Isolated purified cry2 protein was co-illuminate for 20 s at 100 μmol m^−2^s^−1^ blue light *I*_1_ and an increasing fluence rate of green light *I*_2_ as indicated. Normalized absorption spectra are reported. **(B)** Calculated forward rate constant *k*_2_ vs. green light of fluence rate *I*_2_ (red triangles). For each photon fluence rate of panel **(A)** *I*_2_, the rate constant *k*_2_ was calculated by using the three-state algorithm, with the concentration of [FADH^o^] and *k*_1_ (from Figure 2D) as input. We neglected the reoxidation rates *k*_1*b*_ = *k*_2*b*_ = 0 (see text). The linear fit *k*_2_ = σ_2_ *I*_2_ of the data are reported in blue. σ_2_ is the photo-conversion cross section, which resulted σ_2_ = 1.6 × 10^−5^ μmol^−1^ m^2^. From σ_2_ we calculated a quantum yield of ϕ_2_ = 0.027, according to σ_2_ = 2.3 ε_2_ ϕ_2_. For details of calculations see Method Section Three-State Model: Forward Rate *k*_2_ and Quantum Yield ϕ_2_ under Green Light.

**Figure 7:**
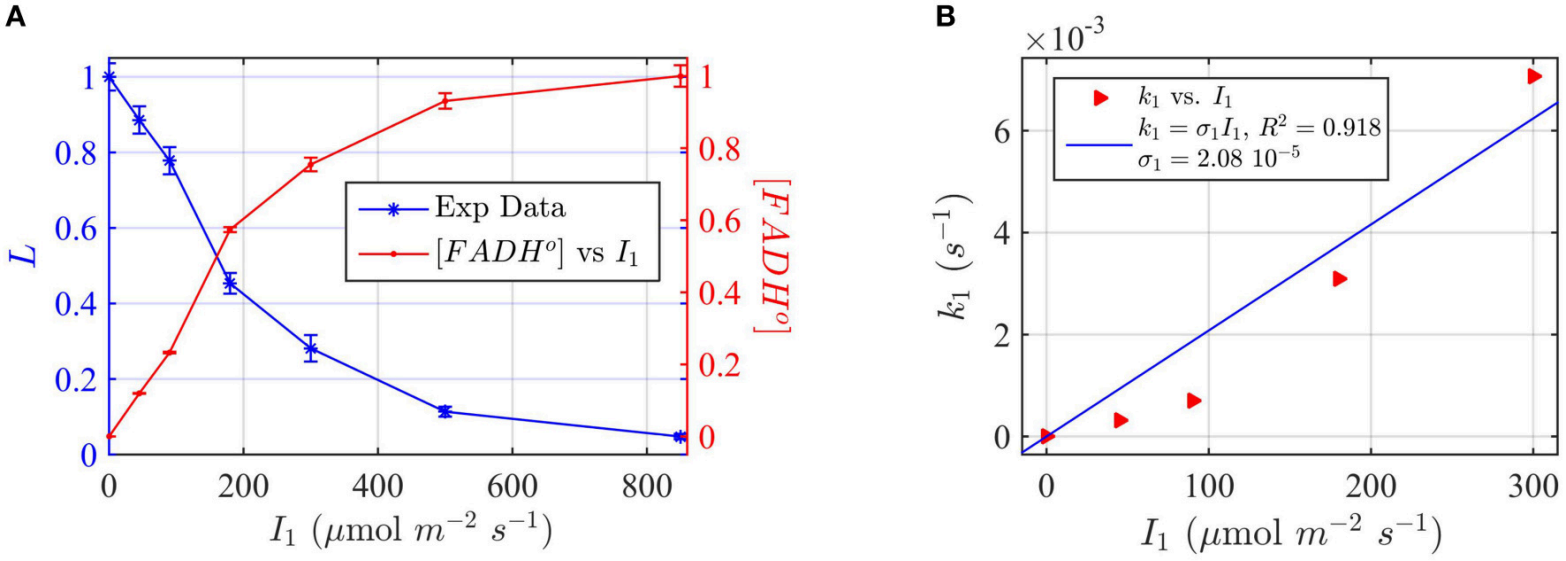
**Modeling ***in vivo*** Cry1 biological activity in blue light. (A)** Double plot of (left, blue vertical axis) of normalized hypocotyl length L, and (right, red vertical axis) [FADH^o^], as a function of the photon fluence rate *I*_1_. The FADH^o^ values were obtained from L by assuming that the hypocotyl length is inversely proportional to FADH^o^ concentration, as explained in Method Section Three-State Model of Cryptochrome Photoreduction. The error bar represents the standard error of the hypocotyl growth measurements for each *I*_1_. **(B)** Rate constant *k*_1_ as a function of the photon fluence rate *I*_1_ (red triangles). The rate constant *k*_1_ was calculated by the two-state model algorithm, with input of the [FADH^o^] concentration, *k*_1*b*_ = 0.0023 s^−1^ and with output the *k*_1_. The blue curve reports the fit of the data, from which a photoconversion cross section of σ_1_ = 2.08 × 10^−5^ μmol^−1^ m^2^ resulted. We calculate from σ_1_ (Method Section Two-State Model for Cryptochrome Photocycle *In vitro*), a quantum yield of ϕ_1_ = 0.014. The *k*_1*b*_ value was taken from the literature (Herbel et al., 2013).

**Figure 8:**
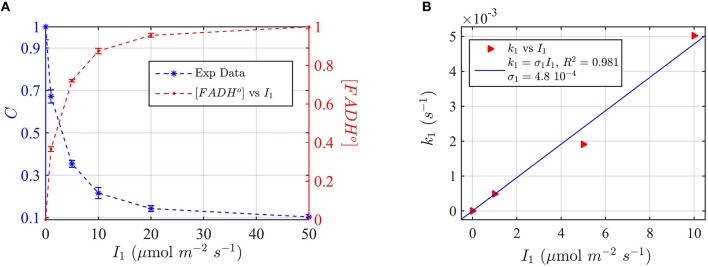
**Modeling ***in vivo*** Cry2 biological activity in blue light. (A)** Double plot of (left, blue vertical axis) normalized protein concentration C, and (right, red vertical axis) [FADH^o^], as a function of photon fluence rate *I*_1_. The FADH^o^ values were obtained from C by assuming that FADH^o^ concentration is inversely proportional to C, as explained in Method Section Three-State Model of Cryptochrome Photoreduction. The error bar represents the standard error of the measurements for each *I*_1_. **(B)** Rate constant *k*_1_ as a function of the photon fluence rate *I*_1_ (red triangles). The rate constant *k*_1_ was calculated by the two-state model algorithm, which inputs the [FADH^*o*^] concentration from panel **(A)**, *k*_1*b*_ = 7.2 × 10^−4^ s^−1^, and outputs *k*_1_. The blue curve reports the linear fit of the data (*k*_1_ = σ_1_ *I*_1_), from which a photoconversion cross section of σ_1_ = 4.8 × 10^−4^ μmol^−1^ m^2^ resulted. From σ_1_ we calculated (as explained in Method Section Two-State Model for Cryptochrome Photocycle *In vitro*), a quantum yield of ϕ_1_ = 0.41, in close agreement with the calculated quantum yield of cry2 *in vitro* (Figure 2D and Table 1). The *k*_1*b*_ value was taken from the literature (Herbel et al., 2013).

**Figure 9:**
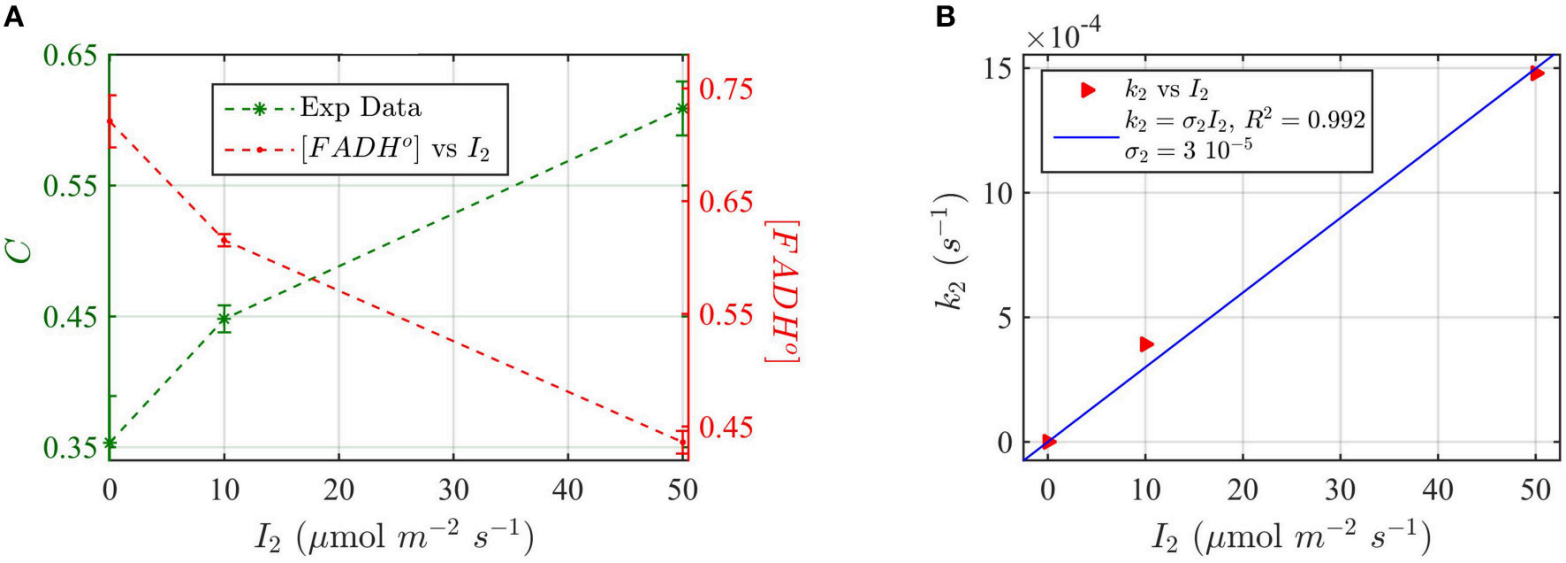
**Three-state Modeling of ***in vivo*** Cry2 biological activity. (A)** Double plot of (left, green vertical axis) normalized protein concentration C, and (right, red vertical axis) [FADH^o^], as a function of the green light fluence rate *I*_2_. Seedlings were co-illuminated at a fluence rate of blue light *I*_1_ = 10 μmol m^−2^s^−1^ and increasing fluence rates of green light (*I*_2_) as shown in the horizontal axis, for 30 min. The FADH^o^ values were obtained from C by assuming that FADH^o^ concentration is inversely proportional to protein concentration, as explained in the Method Section Three-State Model of Cryptochrome Photoreduction. The error bar represents the standard error of the measurements. **(B)** Rate constant *k*_2_ as a function of *I*_2_ (red triangles). The rate constant *k*_2_ was calculated by the three-state model algorithm, which input the [FADH^o^] concentration from panel **(A)**, *k*_2*b*_ = 0.011 s^−1^ (Herbel et al., 2013), *k*_2*b*_ = 7.2 × 10^−4^ s^−1^ (Müller and Ahmad, 2011), and *k*_1_ from Figure 8B. The output is the *k*_2_. The blue curve reports the linear fit of the data (*k*_2_ = σ_2_ *I*_2_), from which a photoconversion cross section of σ_2_ = 3 × 10^−5^ μmol^−1^ m^2^resulted. From σ_2_ we calculated (as explained in Methods Section Two-State Model for Cryptochrome Photocycle *In vitro*), a quantum yield of ϕ_2_ = 0.05, in close agreement with the calculated quantum yield of cry2 *in vitro* (Figure 4D).

*This table summarizes the photoconversion cross sections σ_1_ and σ_2_ expressed, and quantum yields ϕ_1_ and ϕ_2_ of photoconversion of cry1 and cry2 obtained in the course of this study. For comparison purposes both in vitro (Figures 2–7) and in vivo (Figures 8, 9) are shown. τ_1/2_ reports the half-life of the FADH° redox form using the averaged values from fitting the reoxidation data with FADH^o^ (Equation 6) or FAD_ox_ (Equation 7). The in vivo half-lives are taken from the literature (Herbel et al., 2013). The half-life for FADH^−^ to FAD_ox_ reoxidation was considered as 63 s, as suggested from the literature (Müller and Ahmad, 2011)*.

#### Forward photoreduction rate *(k_1_)* and quantum yield (ϕ_1_) of FADox—FADH° conversion

Flavin reduction by light (FAD_ox_ to FADH°) rate constants, and quantum yields, were estimated from experimental results of photoreduction of isolated cryptochrome proteins by using the two-state kinetic model as described in the Method Section Two-State Model: Dark Reoxidation *k*_1*b*_, and Quantum Yield ϕ_1_ under Blue Light. In order to derive the forward rate constant *k*_1_ as a function of photon fluence rate *I*_1_, Figure 2C shows the spectra obtained from cry2 photoreduced at different blue light fluence rates. For each fluence rate *I*_1_ we calculated the rate constant *k*_1_ by using the two-state-based algorithm. This algorithm takes as input the concentration of FADH^o^ (obtained from Figure 2C according to Equation 4), the dark reoxidation rate *k*_1*b*_ previously found (from Figure 2B), and output *k*_1_ according to the two-state model. Figure 2D (red triangles) reports the rate constants *k*_1_ as a function of the blue light fluence rates *I*_1_ (μmol m^−2^s^−1^). The blue curve in Figure 2D is the linear fit of the data (*k*_1_ = σ_1_ *I*_1_) which allows to estimate the photoconversion cross section σ_1_. As can be seen (Figure 2D), the goodness of the fit is excellent (*R*^2^≈1), and resulted in σ_1_ = 1.6 × 10^−4^ μmol^−1^ m^2^. We calculated the quantum yield ϕ_1_ according to σ_1_ = 2.3 ε_1_ ϕ_1_, which resulted ϕ_1_ = 0.14, using the experimentally derived extinction coefficient of ε_1_ = 509.4 mol^−1^ m^2^ (5094 M^−1^ cm^−1^).

By performing the same calculations with FAD_ox_ as input to the algorithm we obtain similar results, with σ_1_ = 2.8 × 10^−4^ μmol^−1^ m^2^ (*R*^2^ = 0.98), and quantum yield of ϕ_1_ = 0.24. This similarity confirms that the two-state kinetic model accurately describes the kinetics of cryptochrome flavin reoxidation under blue light derived experimentally. The average of the two quantum yields is reported in Table 1.

#### Effect of pH on kinetics (*k_1_* and k_1*b*_) of the cry2 photocycle

To further test the validity of the two-state modeling approach under blue light, we evaluate the effect of pH change on the kinetics of the cryptochrome photocycle. At pH 8.2, the efficiency of forward electron transfer is reportedly decreased in *Arabidopsis* cryptochrome-1 (Müller et al., 2014). We accordingly modeled both forward (photoreduction) and back (reoxidation) kinetics of *Arabidopsis* cry2 at pH 8.2, using the same buffer composition and concentration of reducing agent as at pH 7.5 (see Figure 2).

Firstly, AtCry2 samples were photoreduced and returned to darkness (Figure 3A). Spectra were taken at intervals during the dark reoxidation time (*t*_*d*_). Concentration of FADH^o^, obtained from the spectra of Figure 3A, was then plotted as a function of the reoxidation time t_d_ (Figure 3B). The experimental data (red triangles) were fitted with the two-state dark reoxidation model (Equation 7) to find *k*_1*b*_. The resulting reoxidation rate was *k*_1*b*_ = 0.004 s^−1^ (half-life of τ_1∕2_ = 2.7 min), which is very similar to that found at pH 7.5 (Figures 2A,B). By fitting the concentration of FAD_ox_ as a function of the dark reoxidation time with Equation (6), we obtained similar results with *k*_1*b*_ = 0.003 s^−1^ (τ_1∕2_ = 3.2 min, *R*^2^≈1). Therefore, the two states approximation accurately models the experimental results in this case as well. Changes of pH do not affect the reoxidation rate of cry2 protein under these experimental conditions.

We next considered the effect of pH on the forward reaction rate (*k*_1_). Photoreduction of purified cry2 sample was performed at multiple blue light fluence rates (*I*_1_) to obtain decrease at 450 nm and increase at 560 nm indicative of flavin reduction (Figure 3C). From the spectra of Figure 3C we calculated the concentration of FAD_ox_ and FADH^o^. For each photon fluence rate *I*_1_ we calculated the rate constant *k*_1_ by using the concentration of FADH^o^, and *k*_1*b*_ from Figure 3B, as input to the two-state algorithm. Figure 3C reports *k*_1_ as function of *I*_1_ (red triangles), and the linear fit *k*_1_ = σ_1_*I*_1_ (blue curve). The fit gives a photoconversion cross section of σ_1_ = 4.42 × 10^−5^μmol^−1^ m^2^. The quantum yield was then calculated, as explained in the previous section, and resulted ϕ_1_ = 0.038. Using the concentration of FAD_ox_ to find *k*_1_, we obtained similar results (σ_1_ = 5.1 × 10^−5^ μmol^−1^ m^2^, *R*^2^ = 0.98, ϕ_1_ = 0.0435). The quantum yield at pH = 8.2 is therefore one order of magnitude lower than the quantum yield at pH = 7.5.

To summarize the effect of pH change on the cry2 photocycle, our work indicates that the dark reoxidation (*k*_1*b*_) reaction is unaffected by pH change, at least by the change studied here, therefore decrease in quantum yield is due to decreased efficiency in forward light-dependent photoreduction by an order of magnitude. These results are in agreement with prior studies (Müller et al., 2014) validating the accuracy of our model in describing experimental results.

#### Comparison of cry1 and cry2 photoconversion efficiency

Cry1 has been described in the literature as playing a principal role at high blue light intensity, whereas cry2 effects, particularly on photomorphogenesis, are more evident at low blue light intensity (Lin et al., 1998). To provide a further test for the relevance of our kinetic model to experimental findings, we compared the calculated reoxidation rate and quantum yield of cry1 and cry2 flavin reduction *in vitro*, using the two-state model.

Protein samples of cry1 and cry2 were both photoreduced in PBS pH 7.5 with the addition of 5 mM DTT as reductant, since cry1 cannot otherwise be readily reduced under aerobic conditions (Müller and Ahmad, 2011). Dark reoxidation rates for cry1 (Figures 4A,B) and cry2 (Figures 5A,B) were evaluated from spectra taken during the time course of reoxidation by using the two-state model. The reoxidation time t_d_ as a function of the FAD_ox_ concentration is reported in Figures 5B, 6B (red triangles). From the fit (blue curve) the half-life of FADH° to FAD_ox_ interconversion of cry1 resulted τ_1∕2_ = 53 s, while that of cry2 was τ_1∕2_ = 2 min. Similar results are obtained by fitting the decrease in concentration of FADH^o^ with Equation (6) (for cry1 *k*_1*b*_ = 0.016 s^−1^ with *R*^2^≈1, and for cry2 *k*_1*b*_ = 0.0054 s^−1^, with *R*^2^≈1). Once again, the experimental data showed an excellent fit to the model.

Next, the light driven FAD_ox_ to FADH° photoconversion rates were determined experimentally and modeled for cry1 (Figures 4C,D) and cry2 (Figures 5C,D) by using the two-state kinetic model (see Method Section Two-State Model or Cryptochrome Photocycle *in vitro*). Quantum yield calculated for cry1 was ϕ_1_ = 0.043 whereas that for cry2 ϕ_1_ = 0.213.

Similar results were obtained by considering the change in concentration of FADH^o^ to calculate the quantum yield (for cry1 σ_1_ = 4.8 × 10^−5^ μmol^−1^ m^2^ and ϕ_1_ = 0.033 with *R*^2^ = 0.96, and for cry2 σ_1_ = 1.76 × 10^−4^ μmol^−1^ m^2^ and ϕ_1_ = 0.15 with *R*^2^ = 0.97). This means that, under comparable illumination and buffer conditions, cry2 has about 10-fold more efficient response to light than does cry1.

In sum, the two-state kinetic model accurately describes the experimental data for both cry1 and cry2 photoreduction. The obtained quantum yield for cry1, which is 10-fold lower as compared to cry2, is furthermore in good agreement with the biological role of cry2 at lower blue light intensities.

### Three-state model of cryptochrome photoreduction

Under conditions of monochromatic blue light illumination, it is evident from our above results that a two-state model adequately describes the cryptochrome photocycle. This follows from the fact that only two redox states (FADox and FADH°) accumulate to reasonable proportions in blue light, which is absorbed preferentially by FAD_ox_ state and because the FADH° state converts relatively inefficiently to the FADH^−^ redox state (see Figure 1 and Bouly et al., 2007; Burney et al., 2012). Therefore, the contribution of the FADH^−^ state to the equilibrium reached by cryptochrome can be neglected and the two-state model can be applied. However, under natural conditions blue light is only one component of the ambient light environment and there is a higher proportion of UV, turquoise, green, and yellow light (all absorbed by the radical FADH°) than of purely blue light in the spectrum. Therefore, the cryptochrome photocycle is more complex than the simpler “on”—“off” two state conversion model for most photoreceptors, and the three-state kinetic model must be considered.

#### FADH° to FADH^−^ photoconversion

To complete the modeling of the cryptochrome photocycle, we therefore consider the FADH°—FADH^−^ redox state transition (*k*_2_) and its reverse (reoxidation) reaction (*k*_2*b*_). To do so, we take advantage of the spectral properties of the neutral radical (FADH°) flavin redox state, which can absorb green (500—600 nm) as well as blue light. No other redox form of FAD can absorb green light. As can be seen (Figure 1), illumination with green light induces the reduction of FADH° to FADH^−^(*k*_2_ but not *k*_1_). Therefore, the rate constant *k*_2_ can be experimentally determined by assessing the effect of co-illumination of blue plus green light in comparison to blue light alone. Any change in cry2 absorbance induced as a result of co-illumination with green light must necessarily be due to depletion of the neutral radical form (FADH°) of cry2.

To determine the *k*_2_ rate constant for FADH° to FADH^−^ forward light driven photoconversion from the spectral data we have illuminated purified cry2 photoreceptor at a photon fluence rate of blue light *I*_1_ = 100 μmol m^−2^ s^−1^ for 20 s (Figure 6A). This illumination induces formation of the neutral radical redox state. The samples were then co-illuminated with increasing fluence rates of green light (*I*_2_) varying from 0 to 1000 μmol m^−2^ s^−1^ (Figure 6A). Decrease in absorbance at 450 nm and 550 nm with increasing concentrations of green light results from FADH° to FADH^−^ redox state transition.

From the spectra of Figure 6A we calculated the concentration of FADH^o^ and FAD_ox_ by using Equation (4). While the concentration of FADH^o^ decreases with increasing green light fluence rates, FAD_ox_ remains constant (1% variation with respect to the photoreduction with only blue light), meaning that the reoxidation rates can be neglected. By neglecting reoxidation (*k*_1*b*_ = *k*_2*b*_ = 0) we calculated, for each fluence rate *I*_2_, the rate constant *k*_2_ from the three-state algorithm. By taking as input the FADH^o^ values and the rate constant *k*_1_ previously found (Figure 2D), this algorithm outputs *k*_2_ according to Equation (1). Figure 6B reports (red triangles) *I*_2_ as function of *k*_2_, and the linear fit *k*_2_ = σ_2_*I*_2_ (blue curve). The fit provided a photoconversion cross section of σ_2_ = 1.6 × 10^−5^ μmol^−1^ m^2^. As can be seen, also in this case the model (blue line) provides an excellent fit with the data (*R*^2^ = 0.96). The quantum yield for this reaction was calculated as ϕ_2_ = 0.027, which is almost an order of magnitude lower than that of the FAD_ox_ to FADH° interconversion ϕ_1_ = 0.14 (Figure 2D). To confirm that reoxidation can be neglected, we also calculated *k*_2_ with the three-state algorithm by considering the reoxidation rates. For *k*_1*b*_ we used the value previously found in the present manuscript (Figure 2B), and for *k*_2*d*_ = 0.011 s^−1^, a value determined from prior publications (Müller and Ahmad, 2011). We obtained the same results, i.e., ϕ_2_ = 0.027 with σ_2_ = 1.58 × 10^−5^ μmol^−1^ m^2^, confirming that the depletion of FADH^o^ is indeed due to green light. In sum, under conditions of steady state illumination, the receptor will occupy primarily FADH° and FAD_ox_ redox forms, with only a minor contribution from the fully reduced (FADH^−^) redox state (see Supplementary Figure B).

### Kinetic modeling of the cryptochrome photocycle *in vivo*

Ultimately, our goal is to apply the kinetic model, which we used for isolated protein spectra, to predict the equilibrium redox states of cryptochromes adopted in response to illumination *in vivo*. However, it is not possible to obtain direct measurements of the flavin redox state in living plants. Therefore, to apply the kinetic model to cryptochrome photocycle *in vivo*, we make the following assumptions. Firstly, we consider that the simpler two-state model (with only FAD_ox_ and FADH° flavin states) is adequate to describe the cryptochrome photocycle under continuous blue (450 nm) light (Figure 2). This assumption is based on the fact that quantum yield for *k*_2_ is 10-fold lower than for *k*_1_ (Figure 6) and therefore the concentration of FADH° should always be in large excess to that of FADH^−^ (see Figure 1 for interconversion of redox states). Secondly, we make the assumption that biological activity is directly proportional to the concentration of the FADH° flavin state, as indicated by many studies in the literature (reviewed in Chaves et al., 2011). To convert the “readout” for biological activity to FADH^o^ concentration we used Equation (S5) given in the Supplementary Material. Using these two assumptions, we applied the kinetic model depicted in Figure 1 to *in vivo* responses for both cry1 and cry2.

#### Modeling the cry1 photocycle under blue light *in vivo*

In the case of cry1, we chose blue-light dependent inhibition of hypocotyl elongation as a “readout” for biological activity (Ahmad et al., 2002). In this phenotype, seedlings are allowed to grow for several days at different fluence rates of blue light *I*_1_. The length of the hypocotyl (L) is inversely proportional to the blue light fluence rate, and in this case the cryptochrome receptor mediates shortening of the hypocotyl. We chose this property since it is proportional to the photon fluence rate, and we wished to measure a response as closely tied to primary light absorption characteristics at the photoreceptor as possible. To this end we use mutants deficient in phytochrome (*phyAphyB* double mutants) since phytochrome also absorbs blue light and has profound effects on hypocotyl growth, likely by acting downstream of the cryptochrome by an independent mechanism (Ahmad and Cashmore, 1997).

For the experimental procedure, *Arabidopsis* seedlings were grown at different fluence rates of blue light *I*_1_. Hypocotyl length (L) was plotted as a function of blue light fluence rate (Figure 7A).

To apply the two-state kinetic model, we converted hypocotyl length (L) to *in vivo* FADH^o^ concentration, which we double plot as a function of the photon fluence rate in Figure 7A. Figure 7A thus shows our assumption, i.e., that FADH^o^ correlates with the “readout” of biological activity.

For each photon fluence rate *I*_1_ in Figure 7A, we calculated the rate constant *k*_1_, by using the two-state algorithm with input of the FADH^o^ concentration (Figure 7A) and *k*_1*b*_. The dark reoxidation reactions *in vivo* *k*_1*b*_ was taken from previously obtained experimental values (Herbel et al., 2013). The output value was the *k*_1_, as obtained for cry1 *in vitro* (Figure 4). Figure 7B reports *k*_1_ as a function of *I*_1_. The blue curve in Figure 7B reports the liner fit *k*_1_ = σ_1_ *I*_1_, and resulted in a photoconversion cross section of σ_1_ = 2.08 × 10^−5^ μmol^−1^ m^2^. We calculated the quantum yield using the extinction coefficient obtained for cry1 *in vitro*. The quantum yield resulted ϕ_1_ = 0.014, which is in excellent agreement with values obtained from the *in vitro* studies (Figure 4).

#### Modeling the cry2 photocycle under blue light *in vivo*

To model the cry2 photocycle *in vivo*, we have used light-dependent degradation of cry2 as a “readout” for biological function. In the dark, cry2 protein accumulates to high levels in seedlings. However, upon transfer to light, the protein is rapidly degraded within 30 min of the start of illumination (Banerjee et al., 2007; Bouly et al., 2007). This effect relies on conformational change subsequent to light absorption followed by ubiquitination of the receptor, and is therefore directly linked to cry2 primary activation by light and formation of the signaling state. Experiments were performed in phytochrome-deficient mutants (*phyAphyB* double mutants), to avoid potential effects of phytochrome on cry2 degradation. We therefore consider cry2 protein concentration after illumination as a measure for accumulation of the cry2 signaling state and of FADH° accumulation.

In our experiments, we first irradiated dark-grown seedlings at different blue light fluence rates for 30 min. Cry2 protein expression levels were evaluated on Western blots (see Supplementary Figure C) and the signal quantitated by imaging software ImageJ. In this way the protein concentration values C were plotted as a function of the photon fluence rate (Figure 8A).

Also in this case, we converted the “readout” of biological activity, i.e., the cry2 protein concentration C, to FADH° concentration, which we double plot in Figure 8A. We used the FADH^o^ concentration to calculate the photoconversion cross section σ_1_ by plotting *I*_1_ vs. the calculated *k*_1_ (Figure 8B). The value *k*_1d_ for the dark reoxidation reaction of cry2 *in vivo* was already previously obtained experimentally (Herbel et al., 2013). The quantum yield for photoconversion of cry2 was ϕ_1_ = 0.41, again in agreement with the values obtained from *in vitro* studies (Figure 2).

### Three-state model for the cry2 photocycle *in vivo*

Finally, we provide a more comprehensive model of the cry2 photocycle *in vivo* by taking into consideration the third redox state, FADH^−^, which is induced by green light and which had been successfully modeled *in vitro* (Figure 6). We again use the cry2 protein degradation assay as a measure for biological activity (see above), but illuminating with green light (560 nm) in order to induce FADH°—FADH^−^ photoconversion. In this way, the effect of the three redox states on cryptochrome photoconversion could be modeled and compared to the biological activity.

For these experiments, all seedlings were illuminated at a sub-saturating fluence rate of blue light (10 μmol m^−2^s^−1^) for the duration of the light treatments (30 min). Seedlings were in addition co-illuminated with increasing fluence rates of green light *I*_2_. The levels of cry2 protein were analyzed from Western blot images (see Supplementary Figure C for gel image). The cry2 protein concentration was converted in cry2 FADH° concentration and normalized (Figure 9A; increasing concentration of cry2 shows decreased biological activity). For each green light fluence rate *I*_2_, we then calculated the forward rate *k*_2_, by using the three-state model algorithm. We input the normalized FADH^o^ concentration as a function of the photon fluence rate (from Figure 9A), *k*_1_, *k*_1*b*_, and *k*_2*b*_. The *k*_1_ value was as determined in this study (Figure 8), while the *k*_1__*b*_, *k*_2*b*_ values were provided from previous studies (i.e., *k*_1*b*_ = 7.2 × 10^−4^ s^−1^ (Herbel et al., 2013) and *k*_2*b*_ = 0.011s^−1^ (Müller and Ahmad, 2011). The output *k*_2_ was obtained according to Equation (1). Figure 9B reports *I*_2_ as a function of *k*_2_ (red triangles), and the liner fit of the data, *k*_2_ = σ_2_ *I*_2_ (blue curve). From the linear fit, a photoconversion cross section of σ_2_ = 3 × 10^−5^ μmol^−1^ m^2^ was obtained. A quantum yield of ϕ_2_ = 0.05 could be calculated by using the extinction coefficient determined for cry2 *in vitro* (ε_2_ = 254.7 mol^−1^ m^2^, i.e., 2547 M^−1^ cm^−1^). Once again, this value *in vivo* is in close agreement with the quantum yield obtained for cry2 (ϕ_2_) *in vitro* (Figure 6B and Table 1).

## Discussion

This study represents a first attempt to model the cryptochrome photocycle and determine kinetic parameters of relevance to biological function *in vivo*. The method adopted here seeks only to model interconversion of the redox states of cryptochromes (FADox, FADH°, and FADH^−^). It is a much simplified method as compared to global analysis, which has been traditionally used primarily to identify absorbing species from spectra and find reoxidation rates (Müller and Ahmad, 2011). The two-state model studied here is a simple integrable model and the method used here is less computationally expensive than global analysis, and has a similar level of accuracy. We relate absorbance and concentration of two absorbing species (FAD_ox_ and FADH°) by using the Beer-Lambert law, which allows a more accurate fit of the two-state kinetic model to spectra than have been obtained in prior studies (Burney et al., 2009). Most importantly, our method can be readily adapted to estimate quantum yields *in vivo*, which is not the case for global analysis.

Quantum yields have been traditionally obtained at a given photon fluence rate by using different evaluation techniques that were at the same time correlated with receptor photoconversion. Pr/Pfr photoconversion has been followed in phytochromes and related to function in this way, for example (Hermann et al., 1985; Kelly and Lagarias, 1985; Mancinelli, 1988). Quantum yield of *Arabidopsis* cry1 has been calculated in this way for the efficiency of primary electron transfer reactions (Giovani et al., 2003; Müller et al., 2014), which were not, however, related to biological function.

Here, by contrast, we exploit the linear correlation between photon fluence rate and forward rate constant to find the photoconversion cross sections, which we then use to calculate the quantum yields. This method allows us to experimentally find a linear range of intensities where prediction of the two or three states intermediate concentrations can be made.

We have estimated the quantum yield of both cry1 and cry2 *in vitro* from spectra of flavin photoreduction at a range of light intensities. Increasing the buffer pH from 7.5 to 8.2 resulted in a 10-fold decrease in quantum yield of flavin reduction in cry2 derived from the kinetic model, consistent with prior studies showing reduced amplitude of primary electron transfer from flavin under such conditions (Müller et al., 2014). The rate of reoxidation of the FADH° redox state for both cry1 and cry2 was similar under all tested conditions (Figures 2–5), consistent with the dependence of this parameter on the concentration of molecular oxygen (Müller and Ahmad, 2011). In sum, our simple kinetic model accurately describes the redox state transitions of cryptochromes *in vitro* in a manner consistent with effects of known modulating factors.

To model the kinetics of the cryptochrome photocycle *in vivo*, we have made the assumption that biological activity can be used as a measure of FADH° concentration. This follows from numerous studies on the cryptochrome photocycle that have correlated FADH° formation and flavin reduction with biological signaling and activity *in vivo* (Chaves et al., 2011). These include recent studies showing decrease in blue light-dependent biological activity in cry2 (protein degradation assay) in mutants that impair light-dependent radical formation *in vitro* (Li et al., 2011—see also Engelhard et al., 2014). Furthermore, a dose-response curve of cry1 biological activity (hypocotyl growth inhibition) showed decrease in light sensitivity of several orders of magnitude in cry1 photoreduction mutants as compared to the appropriate (expressing similar concentrations of wild type cry1) control seedlings (see supplement in Gao et al., 2015). For the rate of reoxidation from FADH° to FAD_ox_ in our model, we used values derived from *in vivo* studies of the cry1 and cry2 flavin state lifetimes (Herbel et al., 2013). Fitting the kinetic model to biological data, we were able to calculate quantum yields for the biological response *in vivo* which were somewhat similar to the corresponding values calculated from our spectral analysis *in vitro* (see Table 1—compare for example ϕ_1_ values measured for cry1 and cry2 of 0.038 and 0.19 *in vitro* at pH7.5 to ϕ_1_ values obtained *in vivo* of 0.014 and 0.4, respectively).

This similarity (within 2.5-fold) was not a required result for our model to have validity, as many external variables may affect photoreceptor responsivity *in vivo*. For example, the rate constants of the redox state interconversion events (*k*_1_, *k*_2_, *k*_1*b*_, and *k*_2*b*_) could vary greatly *in vivo* through tuning by the cellular environment (Engelhard et al., 2014). Such variability due to cellular environment was also pointed out for the case of the phytochrome (Mancinelli, 1988) where direct measurements of the active state (Pfr) concentration is also not possible *in vivo* (but see Rausenberger et al., 2010). On a more general note, predicted values of the state of photoreceptors calculated from *in vitro* photochemical parameters and the spectral photon flux distribution may vary from the actual ones found *in vivo* as a result of light scattering in whole tissues and shielding by other plant pigments (Mancinelli, 1988). A further problem is the phenomenon of signal amplification through secondary pathways. Indeed, it is possible to derive quantum efficiencies for photoreceptor light sensing *in vivo* that are >1, if the biological readout selected for analysis is subject to significant signal amplification. We address these issues by evaluating phenotypes in etiolated seedlings with a minimum of cell layers and accessory pigments, and also selecting a genetic background (*phyAphyB* double mutant) providing a minimum of signal amplification.

Despite these potential limitations, the fact that quantum yield for biological activity matches so closely with the calculated values for cryptochrome flavin reduction *in vitro* supports our assumption that flavin redox state determines biological activity. This is particularly striking in the case of *k*_2_ for cry2 (response to bichromatic green/blue illumination), for which no other explanation can be reasonably given. Furthermore, qualitative effects *in vivo* such as the relative efficiencies *in vivo* (cry1 as compared to cry2—see below; response to blue as compared to biochromatic blue plus green light) could be clearly determined by this approach.

The quantum yield of cry2 photoconversion (see results summarized in Table 1) is within the range of other photoreceptors such as phytochrome A, which has a photoconversion quantum yield of the order of 0.14 (Gensch et al., 1996) or of the LOV2 domain in phototropins of 0.2 (Kasahara et al., 2002). Cry1 is within the range of quantum yield calculated for LOV1 of 0.026 (Kasahara et al., 2002), which responds at higher blue light intensities. In terms of quantum yields of sensory receptors, it should be considered that cry1 in particular regulates growth processes that occur in full sunlight during de-etiolation and vegetative growth of plants. Therefore, it need not have high quantum yield such as phototropins and phytochrome A, which are specialized for responses at extremely low light intensities (Smith, 1995). To the contrary, too high a photon sensitivity would eliminate the ability to respond to higher intensities of light, as the receptor would reach saturation too quickly. Along these lines the quantum yield of cry2 is around 10-fold higher than cry1, consistent with published effects of cry2 at lower light intensities than for cry1 (Lin et al., 1998).

One of the unique characteristic of the cryptochrome photocycle is the fact that it exists in three states rather than solely as a two states system. The *in vitro* obtained quantum yield for cry2 photoconversion were 0.188 (FADox to FADH°; Figure 2) and 0.027 (FADH° to FADH^−^; Figure 6). This means that under continuous illumination in monochromatic blue light, essentially only the radical (FADH°) redox form should accumulate in response to illumination. This is in distinction to photolyases which undergo full reduction under continuous illumination (Burney et al., 2012). However, under natural conditions of full sunlight, there is a significant contribution from other wavelengths of light, including green light. In particular, in the case of shading under plant canopies, the ratio of green to blue light can be quite high as green light passes through shading leaves whereas blue light is absorbed. Under these conditions, the biological activity of cryptochrome could be considerably modulated by the second (FADH° to FADH^−^) redox state transition, as has indeed been suggested in prior studies (Bouly et al., 2007).

Variations of the three-state photocycle appear to be of general relevance to cryptochrome activation. For instance, in the case of algal cryptochromes (Beel et al., 2012) it appears that the FADH° redox state is the (dark) resting state and the FADH^−^ redox state is the signaling state. Therefore, because the FADH° redox state also absorbs green and red light, algal cryptochromes are activated by UV, blue, green, and red light rather than just blue light such as for plant cryptochromes. Conversely, the drosophila cry appears to adopt just a two-state (FAD_ox_ to FAD^°−^) photocycle, with FAD^°−^ as the signaling state (Berndt et al., 2007). Intriguingly, the avian cry1a, which is thought to be implicated in sensing of the geomagnetic field (Ritz et al., 2000), also appears to adopt a three-state photocycle wherein the FADH^−^ but not the FADH° redox state is the signaling state (Nießner et al., 2013). The kinetic model described in this work should therefore be applicable to all of these cryptochrome photocycles.

In sum, the plant cryptochrome photocycle provides the basis for a rapid and versatile response to the light environment. The receptor can respond within a matter of minutes to changes in the light intensity, even at very high light, by a simple shift in the equilibrium concentration of the FADH° redox state. Because cry1 and cry2 have overlapping functions but the quantum yield of cry2 is 10-fold higher, the combined action of cry1 and cry2 is capable of sensing and responding to light intensity in an almost linear fashion over two orders of magnitude. In this way, the cryptochromes appear to divide the task of responding to the light environment along the lines of what is seen for phytochromes, where the most abundant but photolabile phytochrome (phyA) is specialized for response to dim light whereas stable but less abundant phytochromes (phy B,C,D,E) respond to light at higher intensities (Smith, 1995). Finally, the cryptochromes also have the unique feature of a three-state photocycle, which provides for differential responsivity depending on the wavelength composition. Our model should therefore be useful in unraveling the details of the cryptochrome photocycle in any biological system.

## Author contributions

MA: Designed and performed experiments, wrote paper. JL: Performed experiments. MP: Performed modeling, wrote paper. TR: Assisted modeling. JW: Performed experiments.

## Funding

Funding was from AFOSR (FA9550-14-0-0409).

### Conflict of interest statement

The authors declare that the research was conducted in the absence of any commercial or financial relationships that could be construed as a potential conflict of interest.
