# Supplementary material for: Kinetic Modeling of the Arabidopsis Cryptochrome Photocycle: FADHo Accumulation Correlates with Biological Activity
Source: Front Plant Sci. 2016 Jun 28;7:888. doi: 10.3389/fpls.2016.00888 (PMC4924484; doi:10.3389/fpls.2016.00888)
Supplement: Supplementary file 1 [file DataSheet1.docx]

Supplementary Material

Kinetic modeling of the Arabidopsis cryptochrome photocycle: FADH^o^ accumulation correlates with biological activity.

Maria Procopio 1,2, Justin Link 3, Dorothy Engle 3, Jacques Witczak 2, Thorsten Ritz 1, Margaret Ahmad 2,3*

^1^Department of Physics and Astronomy, University of California, Irvine, CA, USA

^2^UMR 8256 (B2A), IBPS, University of Paris VI, Paris, France

^3^Xavier University, Cincinnati, Ohio, USA

*** Correspondence:** Margaret Ahmad, University of Paris VI, 9 Quai St Bernard, 75005, Paris, France.

margaret.ahmad@umpc.fr

# Analytical solutions of the two-state kinetic model

The analytical solution of the two-state kinetic model reported in Eq. 5 in the main text is straightforward, and gives the concentration of the two redox states as a function on the time t:

$$\begin{matrix} \left[ {FAD}_{ox} \right]\left( t \right)=\frac{{Ck}_{1b}}{k_{1}+k_{1b}}+\frac{Ck_{1}}{k_{1}+k_{1b}}e^{-\left( k_{1}+k_{1b} \right)t} \\ \end{matrix} (S1)$$

$$\begin{matrix} \left[ {FADH}^{o} \right]\left( t \right)=\frac{{Ck}_{1}}{k_{1}+k_{1b}}({1-e}^{-\left( k_{1}+k_{1b} \right)t} ) \\ \end{matrix} (S2)$$

where *C* is the initial concentration of FAD_ox_, i.e. the concentration of Cry at time t=0, before illumination. This condition holds [FADox](t)+[FADH](t)=C at a given time t.

**Forward photoreduction rate (k_1_). Two-state model**

By illuminating cry samples for times shorter that the re-oxidation time, k_1b_=0, in Eq. 2, and the and analytical solutions for this case are:

$$\ln\left( \frac{\left[ {FAD}_{ox} \right]\left( t \right)}{c_{ox}} \right)/t=\ln\left( \frac{{2 A}_{450} \left( t \right)-1}{A_{450}\left( 0 \right)} \right)/t=-k_{1} (S3)$$

$$\frac{\ln\left( 1-\frac{\left[ {FADH}^{o} \right]\left( t \right)}{c_{ox}} \right)}{t}=\frac{\ln\left( 1-\frac{{2 A}_{550}\left( t \right)}{A_{450}\left( 0 \right)} \right)}{t}=-k_{1} (S4)$$

From these equations one can calculate the rate constant k_1_ from the spectra by applying the Eq. 4 reported in the main text.

# From the hypocotyl length L and protein concentration C to FADH^o^ concentration

To convert the length L and protein concentration C in FADH^o^ concentration, we assume that at the maximum length L_max_ or concentration C_max_, (obtained in dark), the [FADH^o^] concentration is zero, [FADH^o^]=0, while at minimum length L_min_ or concentration C_min_ the normalized concentration of [FADH^o^] =1. To perform a change of range values we apply the following formula:

$$y=a+\frac{(x-A)(b-a)}{(B-A)} (S5)$$

which transforms the range of values [A,B] to the new range [a,b]. Thus, A=L_max_ (C_max_), and B=L_min_ (C_min_), *a* is the minimum concentration of FADH^o^, i.e. a=[FADH^o^]=0, and *b* is the maximum concentration, i.e. b=[FADH^o^]=1. In this way a dose-biological response profile can be convened in dose-FADH^o^ concentration profile, which can be used to find quantum yields.

# Supplementary figures

| 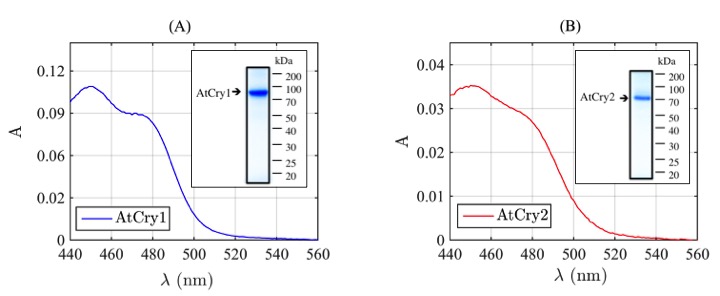  Figure A. Absorption spectra of (A) AtCry1 and (B) AtCry2. The inset represents an SDS-PAGE gel image of the purified protein sample from which the spectra were taken. These spectra were used to calculate the extinction coefficient ε_ox_ at 450 nm. The concentration of cry1 was C=16.99 µM and of cry2 C=6.93 µM. The absorbance at 450 nm is A_450nm_=0.1090 for cry1, and A_450nm_=0.0353 for cry2. For Cry1 the extinction coefficient ε_ox_=6415.5 M^-1^ cm^-1^, and for cry2 ε_ox_=5094 M^-1^ cm^-1^. The path length was d=1 cm. |
| --- |

| 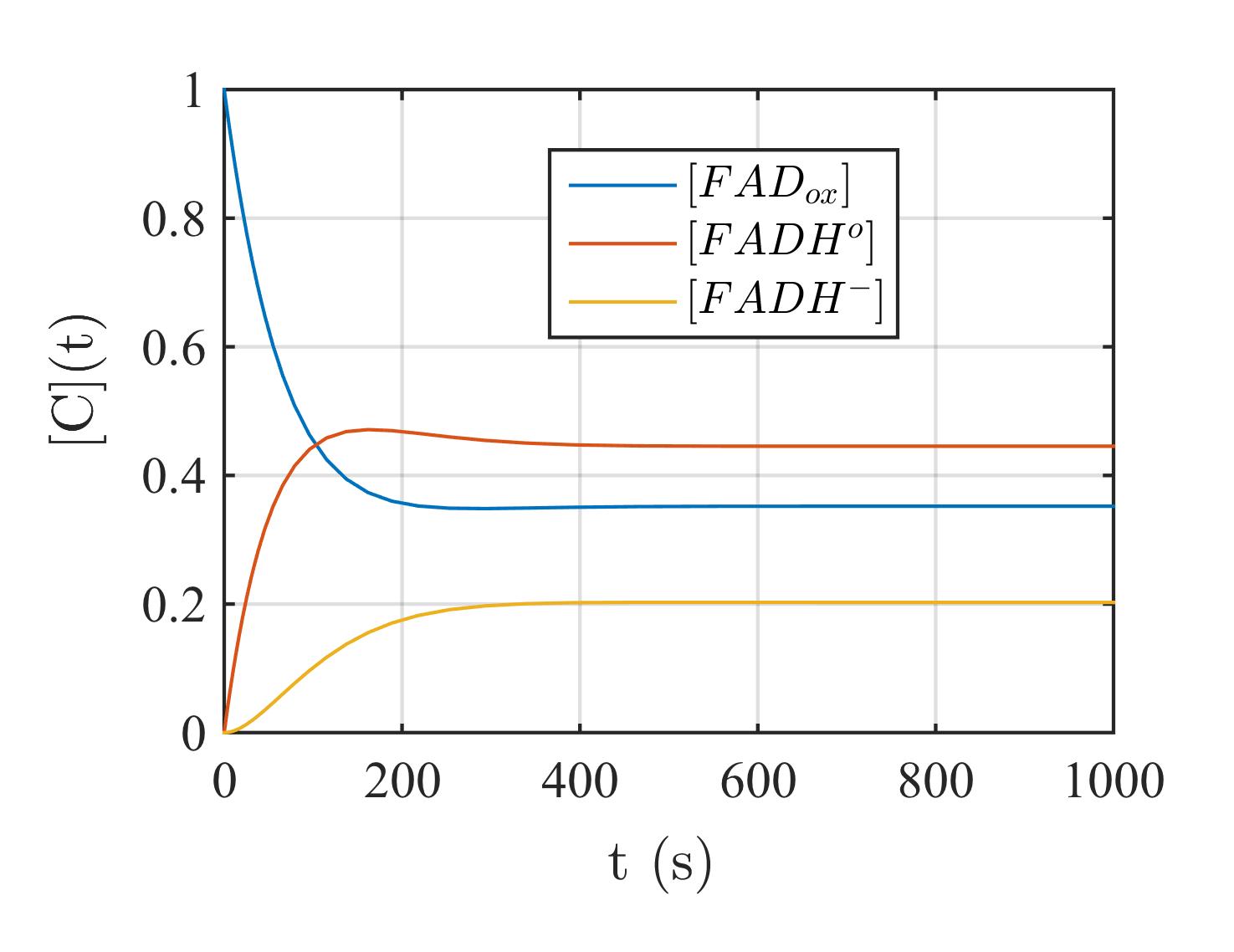 | **Figure B**. **Concentration of FADox, FADH^o^ and FADH^-^ as a function of time, until steady state is reached.** This three-state model simulation corresponds to a co-illumination of AtCRy2 at pH=7.5, with blue light I_1_=100 and green light I_2_ =450 (μmol m^-2^ s^-1^) for 1000 seconds. The rate constants used for the simulation are k_1b_, and k_1_ taken from Fig. 2, and k_2_ from Fig. 6. [C] labels the normalized concentration of the three states as function of time t. |
| --- | --- |

| 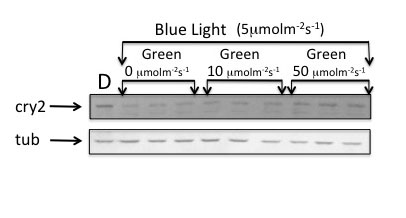  Figure C. Cry2 protein degradation in response to Blue and Green Light. 4 day old etiolated Arabidopsis seedlings were illuminated at the indicated light intensities of blue and green light for 30 minutes prior to harvest into liquid nitrogen. Three independently harvested samples were analyzed per light condition. Extracted proteins were resolved on SDS gels and transferred to nitrocellulose membranes for Western blot analysis as described in Methods. The blot was probed with anti-Cry2 antibody (cry2) showing differential protein levels depending on the light treatments. Blots were probed with Alpha-tubulin antibody (see reference Herbel et al. 2013 reported in the main text) as a loading control. |
| --- |
